# Supplementary material for: Cooperation in alternating interactions with memory constraints
Source: Nat Commun. 2022 Feb 8;13:737. doi: 10.1038/s41467-022-28336-2 (PMC8825791; doi:10.1038/s41467-022-28336-2)
Supplement: Supplementary file 1 — Supplementary Information [file 41467_2022_28336_MOESM1_ESM.pdf]

Supplementary Information

Cooperation in alternating interactions with memory constraints

Peter S. Park[1], Martin A. Nowak[1,2], Christian Hilbe[3]

[1] Department of Mathematics, Harvard University, Cambridge MA 02138, USA
[2] Department of Organismic and Evolutionary Biology, Harvard University, Cambridge MA 02138, USA
[3]Max Planck Research Group Dynamics of Social Behavior, Max Planck Institute for Evolutionary Biology, 24306 Ploen, Germany

Contents

Supplementary Figures 2

Supplementary Note 1: Baseline model 11

Supplementary Note 2: Equilibrium analysis for alternating games 13

Sufficiency of reactive strategies 14
Best responses to memory-1 strategies 16
Classification of memory-1 Nash equilibria 18
Alternating games with implementation errors 21

Supplementary Note 3: Extensions of the baseline model 27

Finitely repeated games 27
Irregular alternation patterns 31
Memory-2 strategies 33
Games in spatial populations 38
The effect of local mutations 40

Supplementary Note 4: Proofs of the analytical results 41

Supplementary References 49

Supplementary Figures

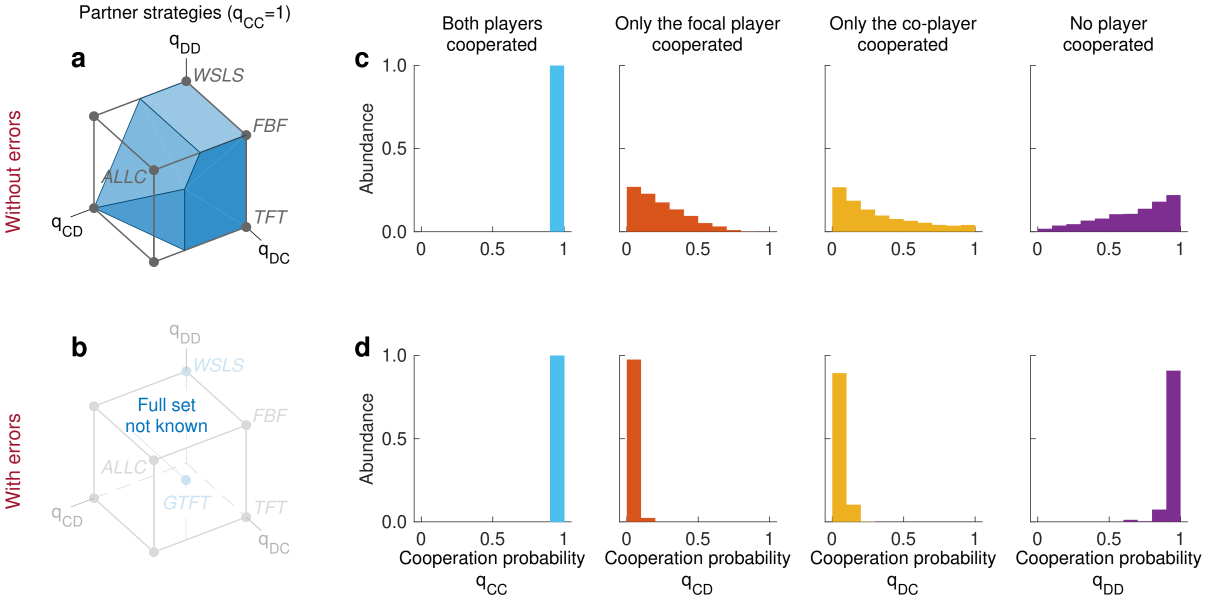

Supplementary Figure 1: Partners in the simultaneous game. Here, we present results analogous to those of **Fig. 4**, but illustrating the outcome of simultaneous games. **a,c,** Without errors, the two models make the same predictions, and also the evolving cooperative strategies are similar. **b,** With errors, there is no theory yet that characterizes all partner strategies of the simultaneous game. It is only known that particular cooperative strategies are stable under certain conditions. For example, *WSLS* is a partner strategy if $b > 2c$, provided the error rate is sufficiently small[1]. Another example of a partner strategy is *GTFT* (defined in the same way as in the alternating game; for reactive strategies, the two games are equivalent[2]). **d,** For simultaneous games with errors, our evolutionary simulations confirm that players predominantly maintain cooperation with *WSLS*. Parameters are the same as in **Fig. 4**.

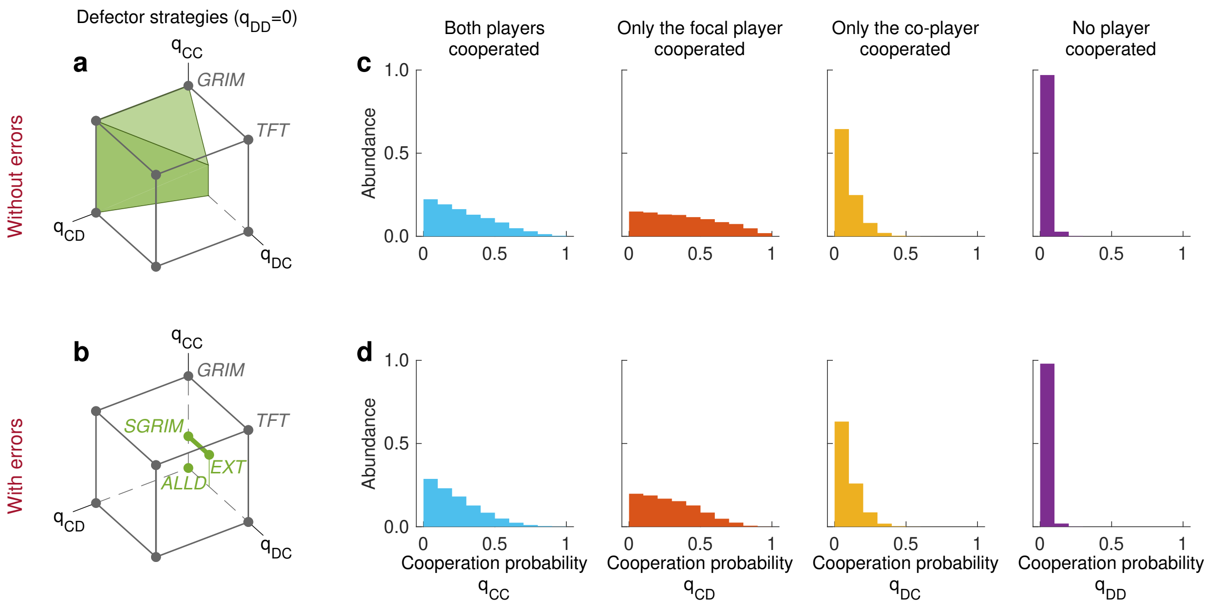

Supplementary Figure 2: Defectors in the alternating prisoner's dilemma. As we have done for partners, we have also characterized all defectors among the memory-1 strategies for the alternating game. **a,b,** We provide explicit conditions for the case without errors and the case with errors, see **Supplementary Note 2**. With errors, there are two classes of defector strategies. First, there is the atomic class of unconditional defection (*ALLD*). Second, there is a line segment that connects a stochastic version of *GRIM* to the strategy *EXT*; the latter is a limiting version of the previously described extortionate strategies[3–7]. **c,d,** For the evolutionary simulations, we use the same parameters as in **Fig. 4**.

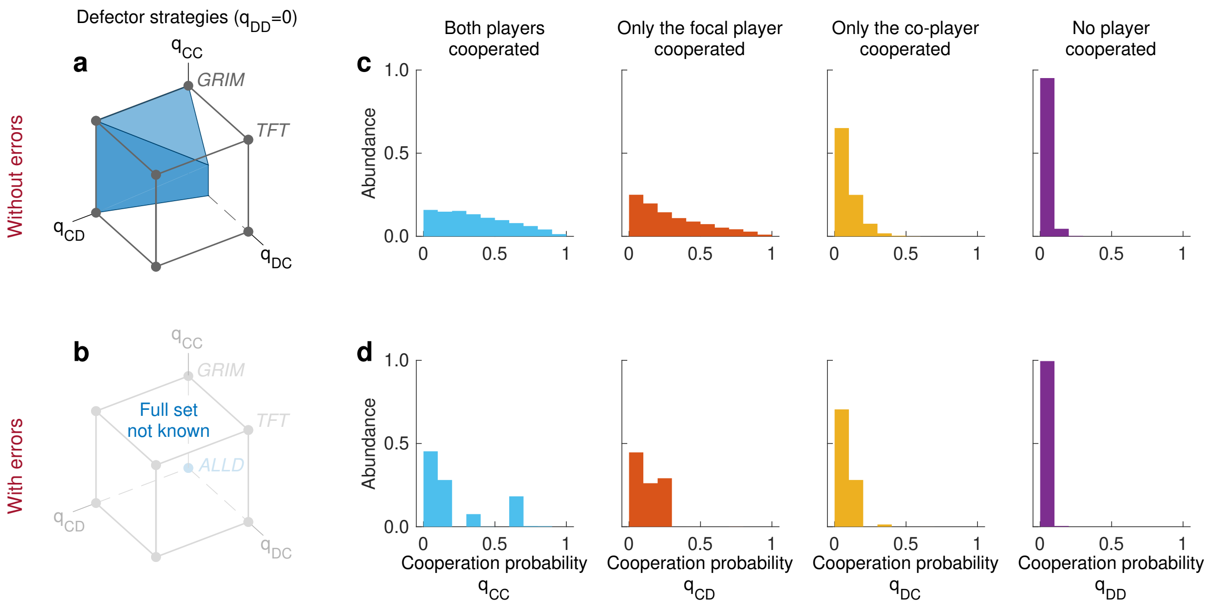

Supplementary Figure 3: Defectors in the simultaneous game. a,b, The figure is analogous to **Supplementary Fig. 2**, but for the case of the simultaneous game instead of the alternating game. For the simultaneous game with errors, there is no complete characterization of defector strategies as of yet. However, it is known that *ALLD* is a Nash equilibrium for all parameter values, because it simply reiterates the Nash equilibrium of the one-shot game. **c,d,** For the evolutionary simulations, we use the same parameters as in **Fig. 4**.

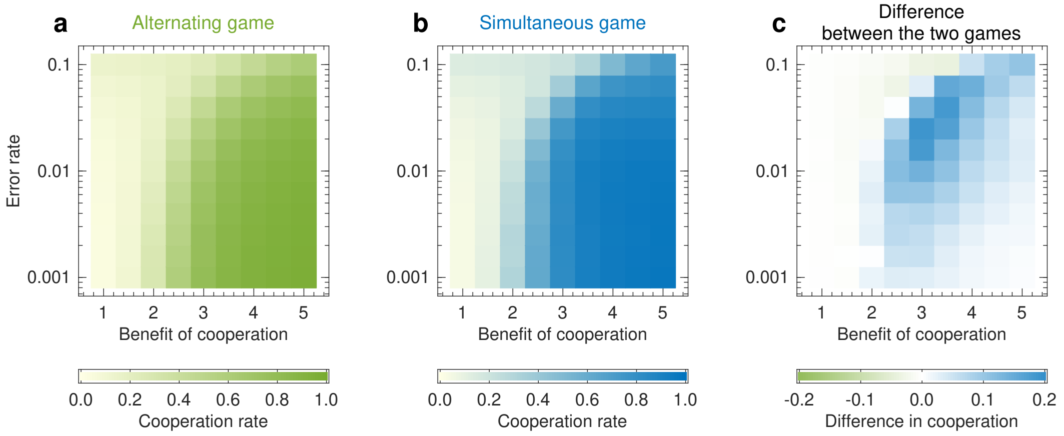

Supplementary Figure 4: Comparing the alternating and the simultaneous game across different error rates and benefit values. We have run further simulations to explore the joint effect of the error rate and the benefit of cooperation, for both the alternating game (**a**) and the simultaneous game (**b**). In both cases, we observe high cooperation rates for high benefit values and sufficiently small error rates. **c,** Here, we plot the difference in cooperation rates between the simultaneous and the alternating game. This difference is small for small benefit values (where defection evolves in both settings). It is also small for large benefits when the error rate is small (for which nearly full cooperation evolves in both settings). In between, for intermediate benefit values and intermediate error rates, the simultaneous game yields systematically more cooperation than the alternating game. Baseline parameters are the same as in **Fig. 5**.

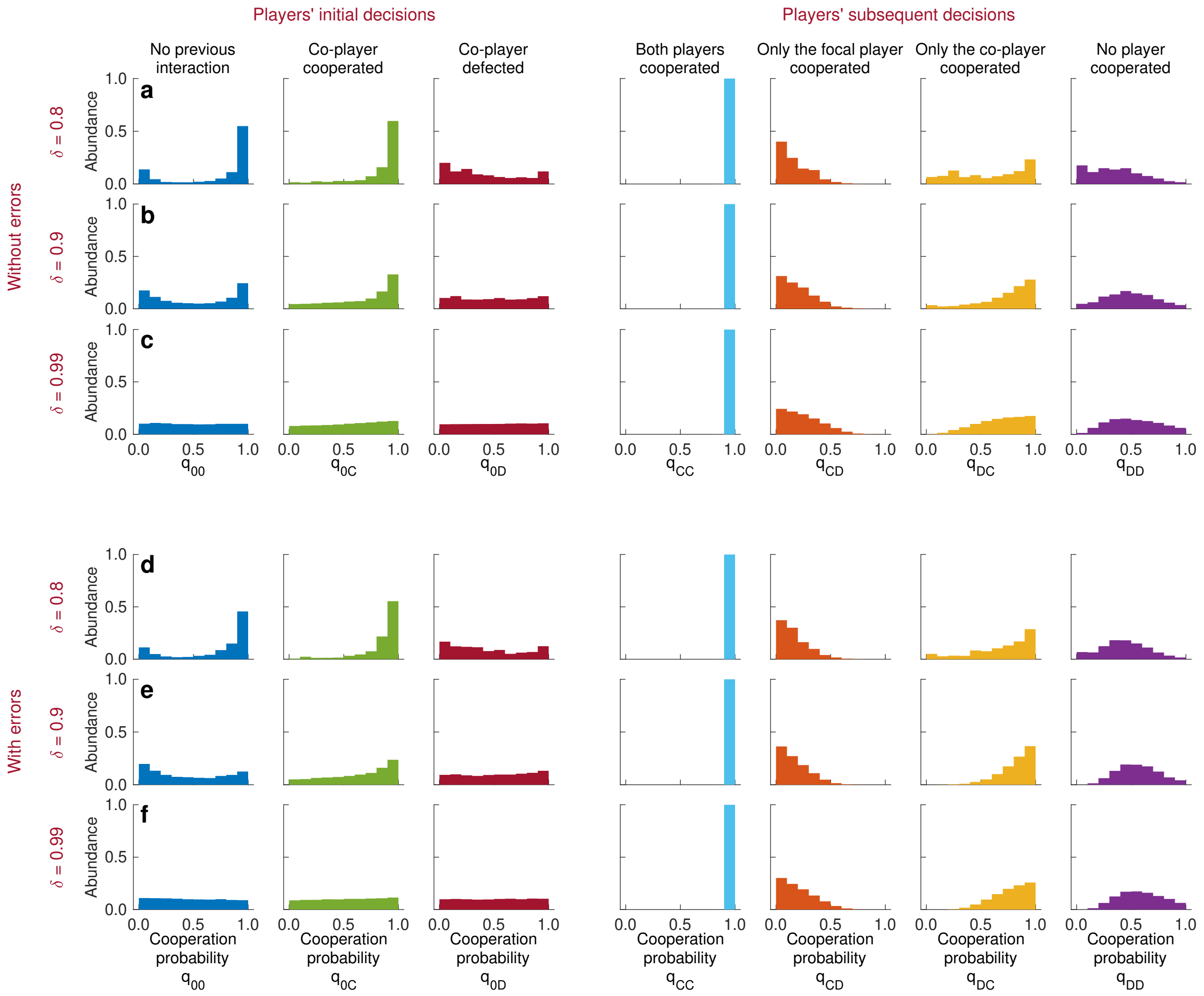

Supplementary Figure 5: Self-cooperative strategies in the finitely repeated alternating game. We explore which strategies the players use to maintain cooperation in finitely repeated alternating games. To this end, we consider two different error scenarios (**a-c:** $\varepsilon = 0$ and **d-f:** $\varepsilon = 0.02$), and three expected game lengths (5, 10, or 100 rounds, corresponding to a continuation probability of $\delta = 0.8$, $\delta = 0.9$, and $\delta = 0.99$). In each case, we run simulations and record those strategies that have a self-cooperation rate of at least 80%. Here, we show the distribution of these strategies. We observe the following regularities: (*i*) The players' first round behavior is only under selection when players interact for a few rounds. For $\delta > 0.9$, the distribution of the respective cooperation probabilities q_{00}, q_{0C}, q_{0D} is comparably flat. (*ii*) The conditional cooperation probabilities for all subsequent rounds generally resemble the evolving strategies of the baseline model. Apart from the parameters varied explicitly, parameters are the same as in **Fig. 4**.

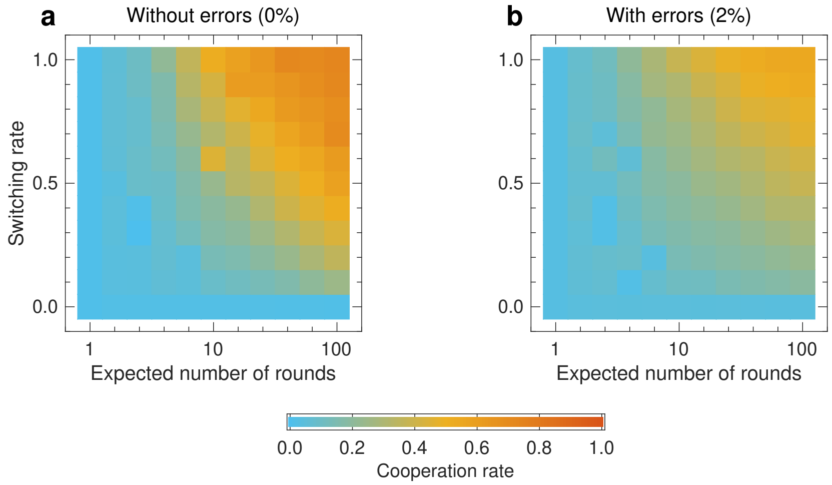

Supplementary Figure 6: Cooperation in the finitely repeated game with irregular alternation patterns. Using the same basic setup as in **Fig. 6**, we have explored how likely players are to cooperate in finitely repeated alternating games with irregular alternation patterns. To this end, we vary (*i*) the expected number of rounds, and (*ii*) the switching rate that measures how strictly players are to alternate. In addition, we consider two scenarios, depending on whether or not players commit implementation errors (**a,b**). As already indicated by **Fig. 6**, players are most likely to cooperate when there are no errors, when the number of rounds is large, and when players move in a strictly alternating fashion.

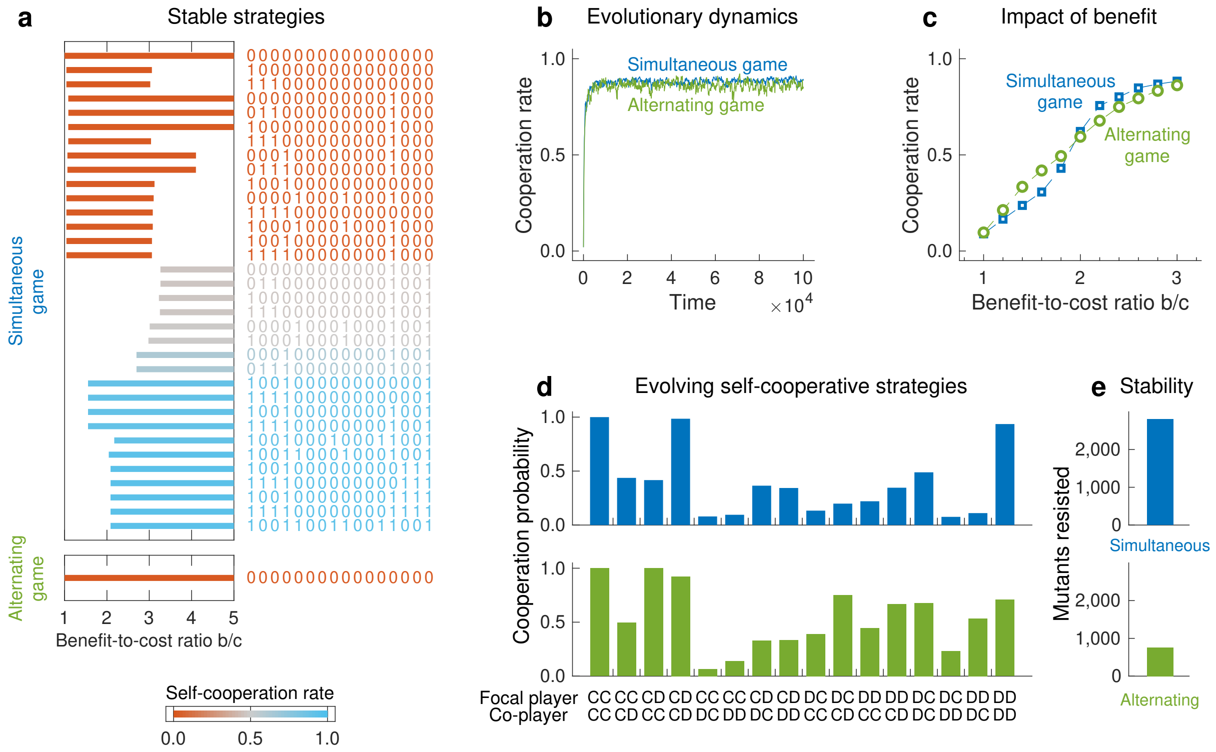

Supplementary Figure 7: Stability and evolutionary dynamics of pure memory-2 strategies. We have also run simulations for (infinitely repeated) simultaneous and alternating games when players have access to all 65,536 memory-2 strategies. These strategies are 16-dimensional vectors that take each player's last two actions into account. **a,** In a first step, we have computed which of these strategies are evolutionarily stable for an error rate of $\varepsilon = 0.02$. Here, we display the respective strategies (encoded by the 16 integers on the right hand side), the range of b/c values for which these strategies are stable (indicated by the length of the lines), and the self-cooperation rates of these strategies (indicated by the color of the respective line). In the simultaneous game, there are many evolutionarily stable strategies, including strategies that yield almost full cooperation. In contrast, in the alternating game, *ALLD* is the only strategy that is evolutionarily stable for a positive range of b/c values. **b,c** Although only the simultaneous game allows for evolutionarily stable cooperation, simulations suggest that alternating games yield similar average cooperation rates. **d,** In a next step, have recorded which strategies the players use to cooperate among themselves (for this simulation we again call a strategy self-cooperative if it yields a cooperation rate of at least 80% against itself). In the simultaneous game, the self-cooperative strategies resemble the previously reported all-or-none strategies[1]. Here, the two players are most likely to cooperate if they both cooperated in the last two rounds, if none of them cooperated in the last two rounds, or if they both cooperated in the last round but defected in the second-to-last round. In the alternating game, the players' conditional cooperation probabilities seem more irregular. **e,** We have also computed how robust self-cooperative strategies are, by recording how many mutant strategies it takes on average to successfully invade into a resident population of self-cooperators. As expected from our evolutionary stability analysis, self-cooperative strategies are more robust in simultaneous games. For details, see **Supplementary Note 3**.

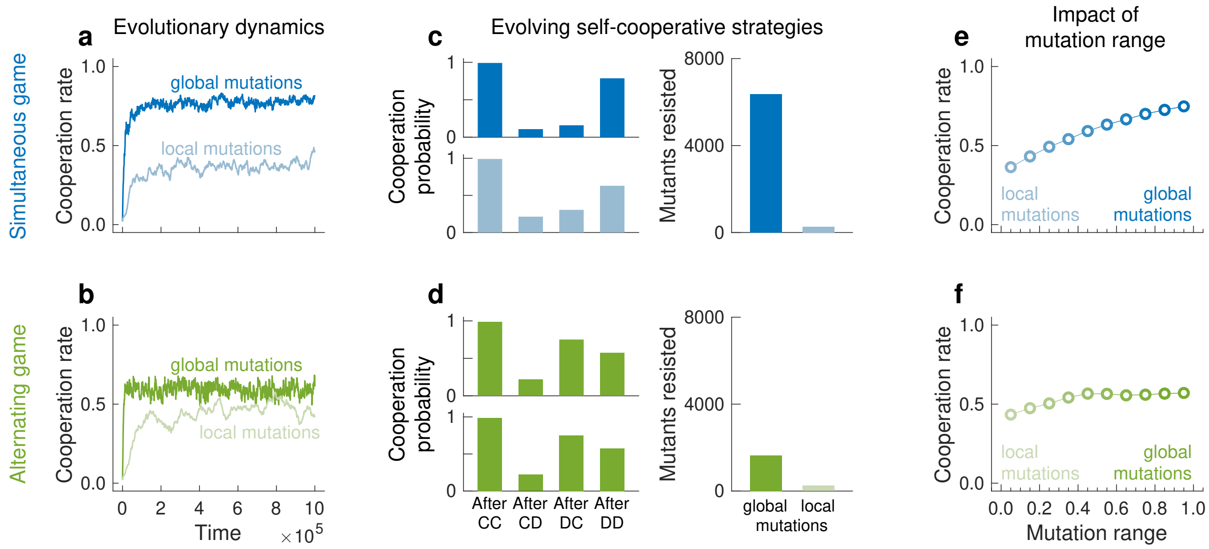

Supplementary Figure 8: Evolutionary dynamics under local mutations. The previous simulations assume that mutations are global: mutant strategies can be arbitrarily far away from the resident strategy. Here we compare this scenario with the case of local mutations, where mutant strategies are required to be in a small neighborhood of the resident strategy. We measure the size of this neighborhood by the mutation range m. The mutation range reflects by how much the mutant's conditional cooperation probabilities are allowed to differ from the resident strategy. Unless noted otherwise, we use $m = 0.05$. **a,b,** In both the simultaneous and the alternating game, local mutations lead to less cooperation. However, the effect is more notable in the simultaneous game. **c,d,** Local mutations do not affect which strategies the players use on average to maintain cooperation. However, they affect how robust these strategies are. Under local mutations, all mutants have approximately the same fitness as the resident. As a result, the evolutionary competition is almost neutral; on average, it thus takes an order of N mutants to invade any given resident population (here, the population size is $N = 100$). **e,f,** We have repeated these simulations for different mutation ranges. The mutation range has a strong effect on cooperation in the simultaneous game (where evolutionarily stable cooperation is possible). It has a comparably weak effect in the alternating game (in which no evolutionarily stable strategy exists that leads to full cooperation).

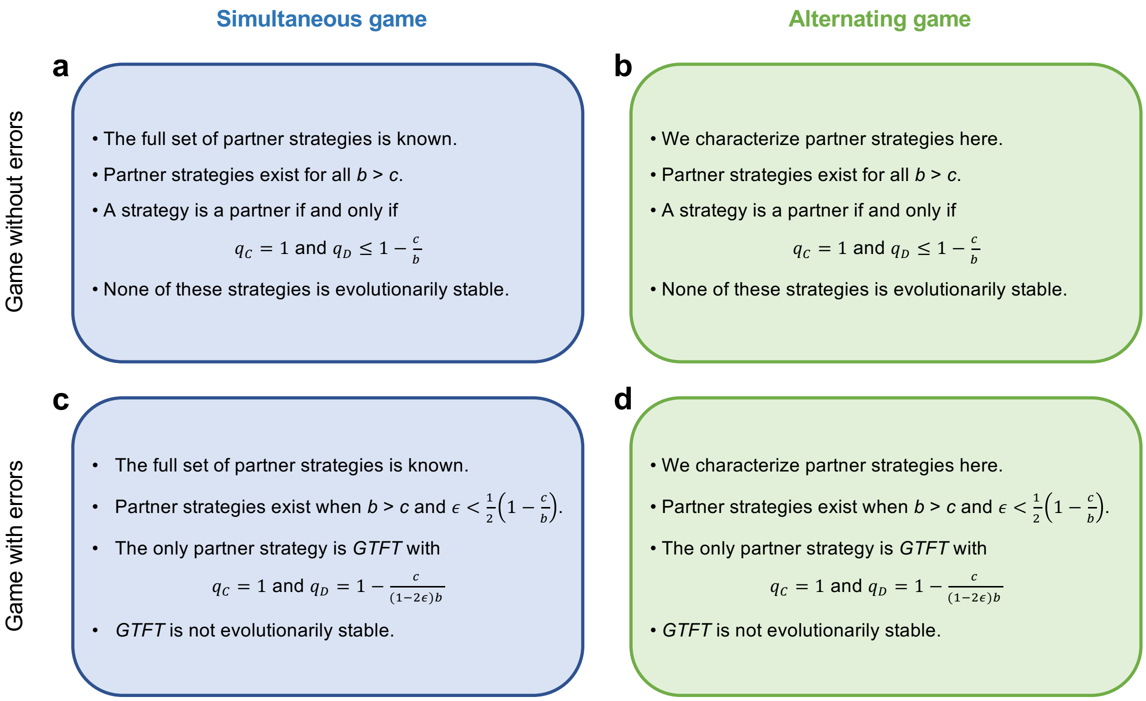

Supplementary Figure 9: A characterization of partners among the reactive strategies. Here, we describe the set of partner strategies within the class of reactive strategies. Reactive strategies are a subset of memory-1 strategies. They consist of two conditional cooperation probabilities, q_C and q_D. The two probabilities describe how a player responds to a co-player's cooperation and defection, respectively. **a,b,** For reactive strategies, the alternating and the simultaneous game lead to the same payoffs[2]. As a result, also the partner strategies coincide in each case. **c,d,** With errors, *GTFT* is the only partner strategy. It can be neutrally invaded by *ALLC*, and hence it is not evolutionarily stable.

We note that in the following, all references to equations refer to the respective equation in the Supplementary Information document; we do not refer to main text equations herein.

Supplementary Note 1: Baseline model

Game setup. In the following, we introduce the model in slightly more general terms than in the main text. We consider two players who interact repeatedly. Each turn, they either decide at the same time whether or not to cooperate (*simultaneous game*), or they decide consecutively, one after the other (*alternating game*). In the former case, players do not know of the other player's decision when making their own decision. In the latter case, player 1 moves first and player 2 learns the outcome before making its own decision. To ensure payoffs are well-defined in both cases, we assume the payoff of an action can be defined based on that particular action alone (that is, the payoff consequences of one player's cooperation does not depend on the co-player's action). This implies that payoffs take the form of the donation game[2]. That is, cooperation (C) implies a cost of $c>0$ to the cooperating player, and it yields a benefit $b>c$ to the co-player. Defection (D) comes with no cost and yields no benefit.

Here we assume the game proceeds indefinitely and future payoffs are not discounted. For such repeated games, we can define the players' payoffs as follows. Let $v_{a_1,a_2}(t)$ denote the probability that the t-th actions of player 1 and player 2 are a_1 and a_2, respectively, with $a_1, a_2 \in \{C, D\}$. Throughout this paper, we assume that for all a_1, a_2 the respective limiting averages are well-defined,

$$v_{a_1,a_2} := \lim_{T\to\infty} \frac{1}{T} \sum_{t=1}^{T} v_{a_1,a_2}(t). \tag{1}$$

These limits are guaranteed to exist, for example, when the two players have finite recall. We collect the four limiting averages defined by Eq. (1) in a vector,

$$\mathbf{v} = (v_{CC}, v_{CD}, v_{DC}, v_{DD}). \tag{2}$$

Each entry corresponds to the probability to observe the respective outcome at a randomly picked time t. Based on these four probabilities, we define the players' payoffs by

$$\begin{aligned} \pi_1 &= b\cdot(v_{CC}+v_{DC}) - c\cdot(v_{CC}+v_{CD}) \\ \pi_2 &= b\cdot(v_{CC}+v_{CD}) - c\cdot(v_{CC}+v_{DC}). \end{aligned} \tag{3}$$

These formulas apply to both the alternating and the simultaneous game (however, the respective limiting averages $\mathbf{v}$ will generally differ, see below).

Memory-1 strategies. We assume players use memory-1 strategies. That is, to decide whether to cooperate in a given round, a player only takes into account each player's most recent decision. Such strategies

can be written as a 4-tuple

$$\mathbf{p} = (p_{CC}, p_{CD}, p_{DC}, p_{DD}). \tag{4}$$

An entry $p_{a\tilde{a}}$ is the probability the focal player cooperates, given that the focal player's last decision was $a \in \{C, D\}$ and that the opponent's last decision was $\tilde{a} \in \{C, D\}$. Such a strategy is *deterministic* if all entries are either zero or one; it is *semi-stochastic* if some but not all entries are between zero and one; and it is *fully stochastic* if all entries are between zero and one.

When both players use memory-1 strategies, the payoffs according to Eq. (3) can be calculated explicitly. To this end, let us consider two players with strategies $\mathbf{p} = (p_{CC}, p_{CD}, p_{DC}, p_{DD})$ and $\mathbf{q} = (q_{CC}, q_{CD}, q_{DC}, q_{DD})$, respectively. The game can be represented as a Markov chain, where the states are the possible combinations of the two players' actions at a given point in time, $\{CC, CD, DC, DD\}$. For the alternating game, the Markov chain's transition matrix is

$$M_A(\mathbf{p}, \mathbf{q}) := \begin{pmatrix} p_{CC}q_{CC} & p_{CC}(1-q_{CC}) & (1-p_{CC})q_{CD} & (1-p_{CC})(1-q_{CD}) \\ p_{CD}q_{DC} & p_{CD}(1-q_{DC}) & (1-p_{CD})q_{DD} & (1-p_{CD})(1-q_{DD}) \\ p_{DC}q_{CC} & p_{DC}(1-q_{CC}) & (1-p_{DC})q_{CD} & (1-p_{DC})(1-q_{CD}) \\ p_{DD}q_{DC} & p_{DD}(1-q_{DC}) & (1-p_{DD})q_{DD} & (1-p_{DD})(1-q_{DD}) \end{pmatrix}. \tag{5}$$

By the Perron-Frobenius Theorem, the vector $\mathbf{v}$ defined by Eq. (2) is an invariant distribution of $M_A(\mathbf{p}, \mathbf{q})$. That is, to compute how often players visit each of the four states, we only need to solve the following linear equation in the unknown $\mathbf{v}(\mathbf{p}, \mathbf{q})$,

$$\mathbf{v}(\mathbf{p}, \mathbf{q}) = \mathbf{v}(\mathbf{p}, \mathbf{q}) \cdot M_A(\mathbf{p}, \mathbf{q}). \tag{6}$$

Based on this invariant distribution, one can then compute payoffs based on Eq. (3),

$$\begin{aligned} \pi(\mathbf{p}, \mathbf{q}) &= b \cdot \Big(v_{CC}(\mathbf{p}, \mathbf{q}) + v_{DC}(\mathbf{p}, \mathbf{q})\Big) - c \cdot \Big(v_{CC}(\mathbf{p}, \mathbf{q}) + v_{CD}(\mathbf{p}, \mathbf{q})\Big) \\ \pi(\mathbf{q}, \mathbf{p}) &= b \cdot \Big(v_{CC}(\mathbf{p}, \mathbf{q}) + v_{CD}(\mathbf{p}, \mathbf{q})\Big) - c \cdot \Big(v_{CC}(\mathbf{p}, \mathbf{q}) + v_{DC}(\mathbf{p}, \mathbf{q})\Big). \end{aligned} \tag{7}$$

For the simultaneous game, payoffs can be computed analogously, but using a different transition matrix[8],

$$M_S(\mathbf{p}, \mathbf{q}) := \begin{pmatrix} p_{CC}q_{CC} & p_{CC}(1-q_{CC}) & (1-p_{CC})q_{CC} & (1-p_{CC})(1-q_{CC}) \\ p_{CD}q_{DC} & p_{CD}(1-q_{DC}) & (1-p_{CD})q_{DC} & (1-p_{CD})(1-q_{DC}) \\ p_{DC}q_{CD} & p_{DC}(1-q_{CD}) & (1-p_{DC})q_{CD} & (1-p_{DC})(1-q_{CD}) \\ p_{DD}q_{DD} & p_{DD}(1-q_{DD}) & (1-p_{DD})q_{DD} & (1-p_{DD})(1-q_{DD}) \end{pmatrix}. \tag{8}$$

In cases in which it is clear which game and which strategies $\mathbf{p}$ and $\mathbf{q}$ are considered (or in case the game and the exact strategies do not matter), we will sometimes write $\mathbf{v}$ and M instead of $\mathbf{v}(\mathbf{p}, \mathbf{q})$, $M_A(\mathbf{p}, \mathbf{q})$, and $M_S(\mathbf{p}, \mathbf{q})$.

We note that in degenerate cases, the solution of $\mathbf{v} = \mathbf{v}M$ does not need to be unique. In that case, the correct invariant distribution $\mathbf{v}$ needs to be derived from the players' actions in the very first round. As an example, consider an alternating game in which both players adopt the strategy *TFT*. The corresponding transition matrix M_A has two absorbing states. The first absorbing state corresponds to indefinite mutual cooperation, and the other corresponds to indefinite mutual defection. Which of these absorbing states is reached (and hence which of the invariant distributions is relevant for the calculation of the players' payoffs) depends on player 1's action in the very first round (when no previous history of actions is yet available). If player 1 cooperates, both players continue to cooperate, and the appropriate invariant distribution is $\mathbf{v} = (1, 0, 0, 0)$. Otherwise, if player 1 defects, the appropriate invariant distribution is $\mathbf{v} = (0, 0, 0, 1)$. We note that if the two *TFT* players interact in a simultaneous game, the respective transition matrix M_S has a third absorbing state. According to that state, players switch between cooperation and defection. In the alternating game this state is no longer absorbing[2], because players now condition their behavior on different past events (as illustrated in **Fig. 1**).

Reactive strategies. An important subset of memory-1 strategies are the so-called reactive strategies. While the behavior of a reactive strategy still depends on the opponent's previous decision, it is independent of the player's own previous decision. Such strategies correspond to those 4-tuples $\mathbf{p} = (p_{CC}, p_{CD}, p_{DC}, p_{DD})$ for which $p_{CC} = p_{DC}$ and $p_{CD} = p_{DD}$. Slightly abusing notation, we denote reactive strategies as 2-tuples $\mathbf{p} = (p_C, p_D)$. The first entry $p_C := p_{CC} = p_{DC}$ is the player's cooperation probability given that the opponent's last decision was to cooperate. The second entry $p_D := p_{CD} = p_{DD}$ is the player's cooperation probability given that the opponent's last decision was to defect. Examples of reactive strategies include *ALLD* $= (0, 0)$, *ALLC* $= (1, 1)$, *TFT* $= (1, 0)$ and *ATFT* $= (0, 1)$.

Supplementary Note 2: Equilibrium analysis for alternating games

In the following, we aim to characterize all symmetric Nash equilibria of the alternating game in the space of memory-1 strategies. In a Nash equilibrium, no player can increase her payoff by unilaterally deviating. To do so, we use an approach that is different from previous approaches for the simultaneous game[9–13]. Our approach involves two steps. First, we show that for any game between two memory-1 players, one can replace one player's strategy by an appropriately chosen reactive strategy without affecting the resulting payoffs. This step is somewhat reminiscent of a result by Press and Dyson[3]. They showed for the simultaneous game that there is no advantage of having a longer memory than the opponent. For alternating games, a stronger result holds. Against a memory-1 opponent, a player can even afford to have a lower memory. It suffices to only remember the opponent's last move and to forget one's own. Second, we show that to find a best response to a given memory-1 strategy, it is sufficient to check it against those reactive strategies that are deterministic. This result implies that one needs to explore only four possible deviations, *ALLD*, *ALLC*, *TFT*, and *ATFT*, as defined above.

Based on these two results, we show that the alternating game allows for three qualitatively different classes of memory-1 equilibria. According to the first two classes, players either mutually cooperate or mutually defect. We refer to the respective strategies as *partners* and *defectors*, respectively. In the last class, players act in such a way that the opponent's payoff is guaranteed to be fixed, irrespective of the opponent's strategy. These strategies have been called *equalizers* in the context of the simultaneous game[3,14].

Sufficiency of reactive strategies

As our first result, we show that when two memory-1 players interact, one player's strategy can be replaced by an appropriate reactive strategy without affecting the game's outcome (all proofs are presented as an appendix in **Supplementary Note 4**). For simplicity, we show this result for the first player. However, because payoffs are independent of the position of the players[2], an analogous result holds for the second player.

Proposition 1 (Sufficiency of reactive strategies when both players use memory-1 strategies)**.**
Consider two memory-1 players with strategies $\mathbf{p}=(p_{CC}, p_{CD}, p_{DC}, p_{DD})$ *and* $\mathbf{q}=(q_{CC}, q_{CD}, q_{DC}, q_{DD})$, *and suppose* $\mathbf{v}(\mathbf{p}, \mathbf{q}) = (v_{CC}, v_{CD}, v_{DC}, v_{DD})$ *is an invariant distribution of the resulting alternating game. We define a reactive strategy* $\tilde{\mathbf{p}} = (\tilde{p}_C, \tilde{p}_D)$ *for player 1 as a solution of*

$$\begin{aligned}(v_{CC}+v_{DC})\,\tilde{p}_C &= v_{CC}\,p_{CC} + v_{DC}\,p_{DC}\\(v_{CD}+v_{DD})\,\tilde{p}_D &= v_{CD}\,p_{CD} + v_{DD}\,p_{DD}.\end{aligned} \tag{9}$$

Then $\mathbf{v}(\tilde{\mathbf{p}}, \mathbf{q}) = \mathbf{v}(\mathbf{p}, \mathbf{q})$. *We call such a strategy* $\tilde{\mathbf{p}}$ *a reactive marginalization of* $\mathbf{p}$ *with respect to* $\mathbf{q}$.

Several remarks are in order.

(a) Intuition for the result. To gain some intuition for **Proposition 1**, let us assume that the strategies $\mathbf{p}$ and $\mathbf{q}$ are such that player 2 both cooperates and defects with positive probability. In that case, $v_{CC}+v_{DC} > 0$ and $v_{CD}+v_{DD} > 0$, and the reactive marginalization of $\mathbf{p}$ with respect to $\mathbf{q}$ is unique,

$$\begin{aligned}\tilde{p}_C &= \frac{v_{CC}}{v_{CC}+v_{DC}}p_{CC} + \frac{v_{DC}}{v_{CC}+v_{DC}}p_{DC}\\\tilde{p}_D &= \frac{v_{CD}}{v_{CD}+v_{DD}}p_{CD} + \frac{v_{DD}}{v_{CD}+v_{DD}}p_{DD}.\end{aligned} \tag{10}$$

That is, to obtain the value of $\tilde{p}_C$, we only need to consider how often the first player cooperates in response to the opponent's cooperation *on average*. To this end, all outcomes in which the first player cooperates are weighted according to how often these outcomes occur in the first place. **Fig. 3** provides an illustration for two particular examples of strategies $\mathbf{p}$ and $\mathbf{q}$.

(b) Proposition 1 only applies to alternating games. To illustrate that the statement is not true for simultaneous games, consider the strategies used in **Fig. 3**, with $\mathbf{p} = (0.9, 0.1, 0.5, 0.3)$ and $\mathbf{q} =$

$(0.8, 0.25, 0.75, 0.2)$. By computing the respective transition matrix M_S according to Eq. (8), and by solving for $\mathbf{v} = \mathbf{v}M_S$, we obtain the invariant distribution $\mathbf{v}(\mathbf{p}, \mathbf{q}) \approx (0.23, 0.21, 0.24, 0.32)$. If we use this expression and formula (10) to compute the unique reactive marginalization of $\mathbf{p}$ with respect to $\mathbf{q}$, we obtain $\tilde{\mathbf{p}} \approx (0.698, 0.220)$. However, the respective invariant distribution of a simultaneous game between $\tilde{\mathbf{p}}$ and $\mathbf{q}$ is $\mathbf{v}(\tilde{\mathbf{p}}, \mathbf{q}) \approx (0.22, 0.23, 0.25, 0.30)$, which is different from $\mathbf{v}(\mathbf{p}, \mathbf{q})$. Hence the simultaneous game between the two memory-1 strategies $\tilde{\mathbf{p}}$ and $\mathbf{q}$ induces a dynamics that is different from the simultaneous game between $\mathbf{p}$ and $\mathbf{q}$.

(c) Non-uniqueness of reactive marginalizations. In some cases of alternating games, a strategy's reactive marginalization is not unique. This happens, for example, if player 1 uses the strategy $\mathbf{p} = (0, 0, 1, 0)$ and the opponent uses *GRIM* $= (1, 0, 0, 0)$. The respective transition matrix M_A according to Eq. (5) has a unique invariant distribution according to which everyone defects, $\mathbf{v} = (0, 0, 0, 1)$. By **Proposition 1** it follows that for *any* reactive strategy $\tilde{\mathbf{p}} = (\tilde{p}_C, 0)$ with $\tilde{p}_C \in [0, 1]$, again $\mathbf{v}$ is an invariant distribution of the game against $\mathbf{q}$. We note however, that one of these reactive strategies, $\tilde{\mathbf{p}} = (1, 0)$, allows for a second invariant distribution, $\mathbf{v} = (1, 0, 0, 0)$. When this reactive marginalization is chosen, we additionally need to require that player 1 defects in the very first round, such that the correct invariant distribution is selected.

The above **Proposition 1** suggests that against a given memory-1 opponent, there is no advantage of choosing a memory-1 strategy instead of a reactive strategy: any payoff a player can achieve with a memory-1 strategy can also be achieved with a reactive strategy. This result holds more generally, even if player 1 has access to more complex strategies.

Proposition 2 (Sufficiency of reactive strategies when only the second player uses a memory-1 strategy)**.** *Consider an alternating game in which the second player uses the memory-1 strategy* $\mathbf{q} = (q_{CC}, q_{CD}, q_{DC}, q_{DD})$ *whereas the first player uses an arbitrary strategy. Denote by* $p_{a_1,a_2}(t)$ *the first player's expected probability to cooperate at time* t *conditional on the players' previous decisions* a_1 *and* a_2*. Suppose the limiting distribution* $\mathbf{v}$ *according to Eq.* (2) *and the following limits on the right hand side exist, such that we can define* $\tilde{p}_C$ *and* $\tilde{p}_D$ *implicitly as a solution of*

$$
\begin{aligned}
(v_{CC}+v_{DC})\,\tilde{p}_C &= \lim_{T\to\infty} \frac{1}{T} \sum_{t=1}^{T} v_{CC}(t)\,p_{CC}(t) + v_{DC}(t)\,p_{DC}(t) \\
(v_{CD}+v_{DD})\,\tilde{p}_D &= \lim_{T\to\infty} \frac{1}{T} \sum_{t=1}^{T} v_{CD}(t)\,p_{CD}(t) + v_{DD}(t)\,p_{DD}(t).
\end{aligned}
\tag{11}
$$

The reactive strategy $\tilde{\mathbf{p}} = (\tilde{p}_C, \tilde{p}_D)$ *so defined satisfies* $\mathbf{v}(\tilde{\mathbf{p}}, \mathbf{q}) = \mathbf{v}$.

The requirements imposed by **Proposition 2** are comparably mild. The existence of the respective limits is guaranteed, for example, if player 1 adopts an arbitrary strategy with finite recall (in some cases, this may again require players to specify their initial actions to make sure the invariant distribution is

well-defined). In the following, we say that those strategies that satisfy the conditions in **Proposition 2** are *generic* with respect to $\mathbf{q}$. That is, generic strategies are those for which one can compute how likely a player is to cooperate on average, conditional on the co-player's previous action. In particular, all memory-1 strategies are generic (given their initial actions are defined). In that special case, the respective definition of $\tilde{\mathbf{p}}$ according to Eqs. (9) and (11) coincide, as one may expect.

We can summarize the results in this section as follows. Given a fixed memory-1 strategy $\mathbf{q}$ for the second player, we can define the following three sets. These sets describe both players' feasible payoffs, given the first player either adopts a reactive strategy, a memory-1 strategy, or a generic strategy, respectively,

$$\begin{aligned} \Pi_R(\mathbf{q}) &:= \Big\{\Big(\pi(\mathbf{p},\mathbf{q}),\ \pi(\mathbf{q},\mathbf{p})\Big) \in \mathbb{R}^2 \ \Big|\ \mathbf{p} \text{ is a reactive strategy}\Big\} \\ \Pi_M(\mathbf{q}) &:= \Big\{\Big(\pi(\mathbf{p},\mathbf{q}),\ \pi(\mathbf{q},\mathbf{p})\Big) \in \mathbb{R}^2 \ \Big|\ \mathbf{p} \text{ is a memory-1 strategy}\Big\} \\ \Pi_G(\mathbf{q}) &:= \Big\{\Big(\pi(\mathbf{p},\mathbf{q}),\ \pi(\mathbf{q},\mathbf{p})\Big) \in \mathbb{R}^2 \ \Big|\ \mathbf{p} \text{ is a generic strategy}\Big\} \end{aligned} \tag{12}$$

Then **Propositions 1** and **2** imply the following.

Corollary 1. *If* $\mathbf{q}$ *is a memory-1 strategy, then* $\Pi_R(\mathbf{q}) = \Pi_M(\mathbf{q}) = \Pi_G(\mathbf{q})$.

That is, against a memory-1 opponent, all payoffs that can either be achieved with a generic strategy, or a memory-1 strategy, can already be achieved with a reactive strategy.

Best responses to memory-1 strategies

In this section, we aim to identify best responses to a given memory-1 strategy. We restrict ourselves to *generic best responses*. A strategy $\mathbf{p}$ is a generic best response to strategy $\mathbf{q}$ if it is generic, and if

$$\pi(\mathbf{p},\mathbf{q}) \geq \pi(\mathbf{p}',\mathbf{q}) \quad \text{for all generic strategies } \mathbf{p}'. \tag{13}$$

By **Proposition 2**, there is always a generic best response in the space of reactive strategies. The following two results simplify the search for a generic best response even further.

Lemma 1. *Consider a reactive player with strategy* $\mathbf{p} = (p_C, p_D)$ *who interacts with a memory-1 opponent with strategy* $\mathbf{q} = (q_{CC}, q_{CD}, q_{DC}, q_{DD})$. *Then, the payoff of the reactive player is given by*

$$\pi(\mathbf{p},\mathbf{q}) = \frac{bq_{DD} - cq_{DD}\cdot p_C + \big((q_{DC}-q_{DD})b-(1-q_{CD})c\big)\cdot p_D + c(q_{CC}-q_{CD}-q_{DC}+q_{DD})\cdot p_C p_D}{1-q_{CD}+q_{DD} - (q_{CC}-q_{CD})\cdot p_C - (q_{DD}-q_{DC})\cdot p_D}. \tag{14}$$

In particular, the payoff of the reactive player depends monotonically on each of its inputs p_C *and* p_D.

The first part of the Lemma gives an explicit formula to compute payoffs. The monotonicity property mentioned in the second part is useful because it allows us to derive the following result.

Proposition 3 (Optimality of deterministic reactive strategies)**.**
Let $\mathbf{q}$ *be some given memory-1 strategy and let* $\mathbf{p}\in[0,1]^2$ *be an arbitrary reactive strategy. Then there is a deterministic reactive strategy* $\mathbf{p}'\in\{0,1\}^2$ *for which* $\pi(\mathbf{p}',\mathbf{q})\geq\pi(\mathbf{p},\mathbf{q})$.

In particular, if $\mathbf{p}$ is a best response to $\mathbf{q}$, then there is at least one deterministic and reactive strategy $\mathbf{p}'$ that yields the same payoff (that is, $\mathbf{p}'$ is also a best response). By combining **Propositions 2** and **3**, we can thus conclude that to find a generic best response to an arbitrary memory-1 strategy, it suffices to consider the four deterministic reactive strategies *ALLD* $=(0,0)$, *ATFT* $=(0,1)$, *TFT* $=(1,0)$, *ALLC* $=(1,1)$.

We can use this observation to characterize all *generic Nash equilibria* among the memory-1 strategies. We say a strategy $\mathbf{q}$ is a generic Nash equilibrium if $\mathbf{q}$ is a generic best reply to itself. By Eq. (7), the payoff of a memory-1 strategy $\mathbf{q}$ against itself is

$$\pi(\mathbf{q},\mathbf{q})=\frac{(1-q_{CC}+q_{DC})q_{DD}}{(1-q_{CC})(1-q_{CD}+q_{DD})+(1-q_{CC}+q_{DC})q_{DD}}\cdot(b-c). \tag{15}$$

Because of **Propositions 2** and **3**, we only need to compare this self-payoff to the payoffs of the four deterministic reactive strategies. By Eq. (14), the respective payoffs are

$$\begin{aligned}
\pi(\mathit{ALLD},\mathbf{q}) &= \frac{q_{DD}}{1-q_{CD}+q_{DD}}\cdot b\\
\pi(\mathit{ATFT},\mathbf{q}) &= \frac{q_{DC}}{1-q_{CD}+q_{DC}}\cdot b-\frac{1-q_{CD}}{1-q_{CD}+q_{DC}}\cdot c\\
\pi(\mathit{TFT},\mathbf{q}) &= \frac{q_{DD}}{1-q_{CC}+q_{DD}}\cdot(b-c)\\
\pi(\mathit{ALLC},\mathbf{q}) &= \frac{q_{DC}}{1-q_{CC}+q_{DC}}\cdot b-c,
\end{aligned} \tag{16}$$

Overall, we obtain the following result.

Theorem 1 (Characterization of generic Nash equilibria)**.**
Let $\mathbf{q}$ *be an arbitrary memory-1 strategy such that the payoffs* (15) *and* (16) *are well-defined. Then* $\mathbf{q}$ *is a generic Nash equilibrium if and only if*

$$\pi(\mathbf{q},\mathbf{q})\geq\max\Big(\pi(\text{ALLD},\mathbf{q}),\ \pi(\text{ATFT},\mathbf{q}),\ \pi(\text{TFT},\mathbf{q}),\ \pi(\text{ALLC},\mathbf{q})\Big) \tag{17}$$

The assumption on the payoffs (15) and (16) to be well-defined is not a major restriction. Those cases in which some of the expressions in Eqs. (15) and (16) cannot be evaluated (for example, when $q_{CC}=1$ and $q_{DD}=0$), correspond to those cases in which the invariant distribution $\mathbf{v}$ according to Eq. (6) is not unique. In that case, one can resolve the ambiguity by defining an initial cooperation probability for the very first round. In this way, all relevant payoffs become well-defined, and condition (17) remains valid.

Classification of memory-1 Nash equilibria

In the following, we use the general characterization provided in **Theorem 1** to give a qualitative classification of all generic memory-1 Nash equilibria. To this end, we first describe three distinct behaviors that can be sustained in equilibrium. These three behaviors correspond to players who mutually cooperate, players who mutually defect, and players who unilaterally fix the co-player's payoff to a fixed level. In line with the previous literature on simultaneous games, we refer to the respective equilibrium strategies as *partners*[11], *defectors*[15], and *equalizers*[14]. Then we show that these three classes of behaviors comprise in fact all Nash equilibria of the alternating game.

Partners. We say a strategy is *self-cooperative* if two players with that strategy obtain the mutual cooperation payoff $b-c$ against each other. For a memory-1 strategy $\mathbf{q}$ to be self-cooperative, Eq. (15) implies that q_{CC} needs to be set to one (if $q_{DD}=0$, the strategy is additionally required to cooperate in the first round). We call a strategy a *partner* if it is self-cooperative and if it satisfies the Nash condition (17). To check whether the Nash condition holds, we note that for any self-cooperative strategy $\mathbf{q}$, the equality $\pi(\mathbf{q},\mathbf{q}) = \pi(\mathit{TFT},\mathbf{q}) = \pi(\mathit{ALLC},\mathbf{q}) = b-c$ holds. Thus, we only need to verify the two remaining inequalities, $\pi(\mathit{ALLD},\mathbf{q}) \le b-c$ and $\pi(\mathit{ATFT},\mathbf{q}) \le b-c$. Based on the respective expressions in Eqs. (16), we conclude that $\mathbf{q}$ is a partner if and only if the following three conditions are satisfied,

$$\begin{aligned} q_{CC} &= 1 \\ (b-c)(1-q_{CD}) &\ge c\,q_{DD} \\ b(1-q_{CD}) &\ge c\,q_{DC}. \end{aligned} \tag{18}$$

These conditions define a 3-dimensional subspace of the memory-1 strategies (see **Fig. 4**). This subspace is non-empty: since $b>c$, all conditions can be met by choosing sufficiently small cooperation probabilities q_{CD}, q_{DC}, q_{DD}. The subspace of partner strategies increases with b and it decreases with c. That is, the more profitable cooperation is, the easier it becomes to satisfy the conditions for being a partner.

Defectors. We call a strategy *self-defective* if two players with that strategy end up with the mutual defection payoff when playing against each other. In particular, a self-defective memory-1 strategy $\mathbf{q}$ needs to set q_{DD} to zero (in case payoffs are not well-defined otherwise, it additionally needs to defect in the first round). We say a self-defective strategy is a *defector* if it additionally satisfies the Nash condition (17). Similar to before, two of the four conditions in Eq. (17) are automatically met because a self-defective strategy satisfies $\pi(\mathbf{q},\mathbf{q}) = \pi(\mathit{ALLD},\mathbf{q}) = \pi(\mathit{TFT},\mathbf{q}) = 0$. Thus, we only need to verify

$\pi(ATFT, \mathbf{q}) \leq 0$ and $\pi(ALLC, \mathbf{q}) \leq 0$. Overall, we obtain the following characterization of defectors,

$$
\begin{aligned}
q_{DD} &= 0 \\
b\, q_{DC} &\leq c\,(1-q_{CD}) \\
(b-c)\, q_{DC} &\leq c\,(1-q_{CC}).
\end{aligned}
\tag{19}
$$

Again, these conditions define a 3-dimensional non-empty subspace of memory-1 strategies (**Supplementary Fig. 2**). The volume of this subspace increases if we either reduce b or increase c.

Equalizers. For simultaneous games, it has been noted that the memory-1 strategies contain a subset of so-called *equalizers*[3,14]. If player 2 adopts such a strategy $\mathbf{q}$, player 1's payoff $\pi(\mathbf{p}, \mathbf{q})$ is a constant, independent of player 1's strategy. Obviously, if both players choose an equalizer strategy, the resulting strategy profile is a Nash equilibrium. In that case, no player can get a different payoff – let alone a larger payoff – by unilaterally deviating.

In the following we aim to identify equalizers in the context of alternating games. That is, we ask which strategies player 2 can use to make sure that player 1's payoff is independent of player 1's strategy. As a minimum requirement, player 2's strategy $\mathbf{q}$ needs to enforce the same payoff upon all co-players with reactive and deterministic strategies, such that

$$
\pi(ALLD, \mathbf{q}) = \pi(ATFT, \mathbf{q}) = \pi(TFT, \mathbf{q}) = \pi(ALLC, \mathbf{q}). \tag{20}
$$

This yields three equations in the four unknown entries of $\mathbf{q}$. By using Eqs. (16) to solve $\pi(ALLD, \mathbf{q}) = \pi(TFT, \mathbf{q})$ for q_{CD} and $\pi(ALLC, \mathbf{q}) = \pi(TFT, \mathbf{q})$ for q_{DC}, we obtain

$$
\begin{aligned}
q_{CD} &= \frac{b\, q_{CC} - c\,(1+q_{DD})}{b-c} \\
q_{DC} &= \frac{b\, q_{DD} + c(1-q_{CC})}{b-c}.
\end{aligned}
\tag{21}
$$

Given these two relations hold, one can verify that the last relation $\pi(ATFT, \mathbf{q}) = \pi(TFT, \mathbf{q})$ holds automatically. Conversely, suppose a memory-1 strategy $\mathbf{q}$ satisfies these two conditions. Because these conditions imply Eq. (20) and because the payoffs of a reactive player $\mathbf{p} = (p_C, p_D)$ are monotonic in p_C and in p_D (due to **Lemma 1**), it follows that any reactive strategy obtains the same payoff against $\mathbf{q}$. Moreover, because generic strategies can be replaced by reactive strategies (**Proposition 1**), it follows that any generic strategy obtains the same payoff against $\mathbf{q}$. We conclude that equalizers are exactly those strategies that satisfy Eq. (21). In particular, equalizers correspond to a 2-dimensional subspace of memory-1 strategies.

The following technical result allows us to show that the three above classes of partners, defectors, and equalizers are in fact all generic Nash equilibria within the space of memory-1 strategies.

Lemma 2. *Consider a memory-1 strategy* $\mathbf{q} = (q_{CC}, q_{CD}, q_{DC}, q_{DD})$ *that is a generic Nash equilibrium, and let* $\tilde{\mathbf{q}} = (\tilde{q}_C, \tilde{q}_D)$ *denote its reactive marginalization with respect to itself.*

1. *If* $\tilde{\mathbf{q}}$ *is fully stochastic, then* $\mathbf{q}$ *is an equalizer.*
2. *If* $\tilde{\mathbf{q}}$ *is semi-stochastic or deterministic, then* $\mathbf{q}$ *is either a partner or a defector.*

We can summarize the previous results as follows.

Theorem 2 (Classification of generic Nash equilibria)**.**
A memory-1 strategy $\mathbf{q} = (q_{CC}, q_{CD}, q_{DC}, q_{DD})$ *is a generic Nash equilibrium if and only if it is either a partner, a defector, or an equalizer: that is, if and only if it meets the conditions* (18), (19)*, or* (21).

Comparing **Theorem 2** for the alternating game with the respective classification of equilibrium outcomes in the simultaneous game[10–12] yields the following two insights:

1. The three classes that we have identified, partners, defectors, and equalizers, also exist in the simultaneous game. In fact, even the respective equilibrium conditions are identical: a memory-1 strategy $\mathbf{q} = (q_{CC}, q_{CD}, q_{DC}, q_{DD})$ is a partner, a defector, or an equalizer in the alternating game if and only if it is a a partner, defector, or equalizer in the simultaneous game (assuming that the game parameters b and c are the same).

2. However, the simultaneous game allows for one additional class of equilibrium strategies, called *self-alternators*[12]. When two self-alternators interact, they cooperate in turns: one player unilaterally cooperates in one round, and the other player unilaterally cooperates in the next. To be a Nash equilibrium for the simultaneous game, self-alternators need to have the form[12]

$$q_{CC} \leq \frac{2c}{b+c}, \quad q_{CD} = 0, \quad q_{DC} = 1, \quad q_{DD} \leq \frac{b-c}{b+c}. \tag{22}$$

   However, according to **Theorem 2**, strategies that satisfy the conditions in (22) do not give rise to a Nash equilibrium in the alternating game.

   The intuition is easy to convey with an example. To this end, let us consider the memory-1 strategy $\mathbf{q} = (0, 0, 1, 1/3)$ which satisfies conditions (22) for all games with $b > 2c$. In the simultaneous game, two players with strategy $\mathbf{q}$ reliably learn to cooperate in turns irrespective of their first-round behavior. This is illustrated by the following sample path (an asterisk indicates a decision that is partly due to chance). In this path, players reliably alternate from the fourth round onwards,

**Player 1**	C	D	D*	C*	D	C	D	C	D	…
**Player 2**	C	D	D*	D*	C	D	C	D	C	…

   In the alternating game, the same first-round behavior gives rise to a more irregular trajectory,

**Player 1**	C		D		D*		C*		D		D*		C		D		D*		…
**Player 2**		C		D		D*		C		D		C*		D		D*		C*	…

Here, there are always two consecutive instances of cooperation, which leads both players to defect on their next turn (because $q_{CC} = q_{CD} = 0$). Once both players have defected, it may take several turns for one player to re-start cooperation (because $q_{DD} = 1/3$). As a result, the limiting average payoff in the alternating game is only $(b-c)/3$. If one of the players were to switch to *ATFT*, the resulting payoff is $\pi(\mathit{ATFT}, \mathbf{q}) = (b-c)/2 > (b-c)/3$. Hence, $\mathbf{q}$ is unstable. We conclude that strategies of the form (22) are no longer a Nash equilibrium in the alternating game because they no longer induce a stable pattern of alternating cooperation.

Alternating games with implementation errors

In the following, we explore how equilibrium behavior is affected by noise. To this end, we assume the players' actions are subject to implementation errors or trembling hand errors,[16]. That is, each time a player wishes to cooperate, there is some probability ε that the player defects by mistake, with $0 < \varepsilon < 1/2$. Conversely, each time a player wishes to defect, she may cooperate with the same probability. Under this assumption, a player with strategy $\mathbf{p}$ employs an *effective strategy* $\mathbf{p}^\varepsilon$, with

$$\mathbf{p}^\varepsilon = \varphi^\varepsilon(\mathbf{p}) := (1-\varepsilon)\mathbf{p} + \varepsilon(\mathbf{1}-\mathbf{p}) = \varepsilon + (1-2\varepsilon)\mathbf{p}. \tag{23}$$

This transformation maps memory-1 strategies $\mathbf{p} \in [0,1]^4$ to noisy memory-1 strategies $\mathbf{p}^\varepsilon \in [\varepsilon, 1-\varepsilon]^4$. It has two useful properties. First, it is bijective, with the inverse function being defined by

$$\big(\varphi^\varepsilon(\mathbf{p}^\varepsilon)\big)^{-1} = \frac{\mathbf{p}^\varepsilon - \varepsilon}{1-2\varepsilon}. \tag{24}$$

Second, the transformation is monotonic: For any previous round's outcome $(a, \tilde{a}) \in \{C, D\}^2$ and for any two memory-1 strategies $\mathbf{p}$ and $\mathbf{q}$, we have $p_{a\tilde{a}} < q_{a\tilde{a}}$ if and only if $p^\varepsilon_{a\tilde{a}} < q^\varepsilon_{a\tilde{a}}$. That is, if player 1's nominal strategy is more cooperative than player 2's, then the same is true for the respective effective strategies. A few examples of effective strategies are

$$\begin{array}{llll} \mathit{ALLD}^\varepsilon = (\varepsilon, \varepsilon) & \mathit{ATFT}^\varepsilon = (\varepsilon, 1-\varepsilon) & \mathit{GRIM}^\varepsilon = (1-\varepsilon, \varepsilon, \varepsilon\,\varepsilon) \\ \mathit{ALLC}^\varepsilon = (1-\varepsilon, 1-\varepsilon) & \mathit{TFT}^\varepsilon = (1-\varepsilon, \varepsilon) & \mathit{FBF}^\varepsilon = (1-\varepsilon, \varepsilon, 1-\varepsilon, 1-\varepsilon). \end{array} \tag{25}$$

In particular, even if the nominal strategy is deterministic, the corresponding effective strategy is fully stochastic. For an alternating game with errors between two memory-1 strategies $\mathbf{p}$ and $\mathbf{q}$ we can define the resulting transition matrix, the invariant distribution, and the payoffs based on the respective quantities for the game without errors, given by Eqs. (5) – (7). This yields

$$M_A^\varepsilon(\mathbf{p}, \mathbf{q}) := M_A(\mathbf{p}^\varepsilon, \mathbf{q}^\varepsilon), \quad \mathbf{v}^\varepsilon(\mathbf{p}, \mathbf{q}) := \mathbf{v}(\mathbf{p}^\varepsilon, \mathbf{q}^\varepsilon), \quad \pi^\varepsilon(\mathbf{p}, \mathbf{q}) := \pi(\mathbf{p}^\varepsilon, \mathbf{q}^\varepsilon). \tag{26}$$

Since $\varepsilon > 0$, each entry of $M_A(\mathbf{p}^\varepsilon, \mathbf{q}^\varepsilon)$ is positive. Hence, the unique invariant distribution and the resulting payoffs are now well-defined irrespective of the players' actions in the first round.

In the following, we aim to characterize all equilibria among the memory-1 strategies for the alternating game with errors. We follow the same approach as before. In analogy to **Propositions 1** and **2**, the following shows that games between a generic player and a memory-1 player can be reduced to a game between a reactive player and a memory-1 player.

Proposition 4 (Sufficiency of reactive strategies in games with errors)**.**
Consider an alternating game with a positive error rate $0<\varepsilon<1/2$*, and suppose the second player uses the memory-1 strategy* $\mathbf{q}$.

1. *Suppose the first player uses the memory-1 strategy* $\mathbf{p}=(p_{CC},p_{CD},p_{DC},p_{DD})$ *and the resulting invariant distribution is given by* $\mathbf{v}^{\varepsilon}(\mathbf{p},\mathbf{q})=(v^{\varepsilon}_{CC},v^{\varepsilon}_{CD},v^{\varepsilon}_{DC},v^{\varepsilon}_{DD})$*. Define the reactive marginalization of* $\mathbf{p}$ *with respect to* $\mathbf{q}$ *as the unique reactive strategy* $\tilde{\mathbf{p}}=(\tilde{p}_C,\tilde{p}_D)$ *for which*

$$\begin{aligned}\tilde{p}_C &= \frac{1}{1-2\varepsilon}\left(\frac{v^{\varepsilon}_{CC}\,p^{\varepsilon}_{CC}+v^{\varepsilon}_{DC}\,p^{\varepsilon}_{DC}}{v^{\varepsilon}_{CC}+v^{\varepsilon}_{DC}}-\varepsilon\right)\\ \tilde{p}_D &= \frac{1}{1-2\varepsilon}\left(\frac{v^{\varepsilon}_{CD}\,p^{\varepsilon}_{CD}+v^{\varepsilon}_{DD}\,p^{\varepsilon}_{DD}}{v^{\varepsilon}_{CD}+v^{\varepsilon}_{DD}}-\varepsilon\right).\end{aligned} \tag{27}$$

   Then, the reactive marginalization satisfies $\mathbf{v}^{\varepsilon}(\tilde{\mathbf{p}},\mathbf{q})=\mathbf{v}^{\varepsilon}(\mathbf{p},\mathbf{q})$.

2. *Suppose the first player uses an arbitrary strategy such that* $p^{\varepsilon}_{a_1,a_2}(t)\in[\varepsilon,1-\varepsilon]$ *is the player's conditional probability to cooperate at time* t *if the previous outcome is* $(a,\tilde{a})\in\{CC,CD,DC,DD\}$*. Let* $\mathbf{v}(t)=\big(v_{CC}(t),v_{CD}(t),v_{DC}(t),v_{DD}(t)\big)$ *be the resulting probability distribution for the player's actions at time* t*. We assume the following limiting averages to exist,*

$$\begin{aligned}v_{a\tilde{a}} &:= \lim_{T\to\infty}\frac{1}{T}\sum_{t=1}^{T}v_{a\tilde{a}}(t)\quad \textit{for all } a\in\{C,D\},\tilde{a}\in\{C,D\}.\\ \tilde{p}^{\varepsilon}_{\tilde{a}} &:= \lim_{T\to\infty}\frac{1}{T}\sum_{t=1}^{T}\frac{v_{C\tilde{a}}(t)\,p^{\varepsilon}_{C\tilde{a}}(t)+v_{D\tilde{a}}(t)\,p^{\varepsilon}_{D\tilde{a}}(t)}{v_{C\tilde{a}}+v_{D\tilde{a}}}\quad \textit{for all } \tilde{a}\in\{C,D\}.\end{aligned} \tag{28}$$

   We define the reactive marginalization $\tilde{\mathbf{p}}=(\tilde{p}_C,\tilde{p}_D)$ *of player 1's strategy with respect to* $\mathbf{q}$ *by*

$$\tilde{p}_C=\frac{1}{1-2\varepsilon}\left(\tilde{p}^{\varepsilon}_C-\varepsilon\right)\quad \textit{and}\quad \tilde{p}_D=\frac{1}{1-2\varepsilon}\left(\tilde{p}^{\varepsilon}_D-\varepsilon\right). \tag{29}$$

   Then, this reactive marginalization satisfies $\mathbf{v}^{\varepsilon}(\tilde{\mathbf{p}},\mathbf{q})=\mathbf{v}$.

Both results follow in a straightforward manner from the respective results on alternating games without errors, by applying **Propositions 1** and **2** to the players' effective strategies. In a similar way, we can also generalize **Proposition 3**. To this end, we say a strategy is generic with respect to the opponent strategy and the error rate if the respective limits in Eq. (28) exist. In particular, all strategies with finite recall are

generic. In addition, we say a strategy $\mathbf{p}$ is a generic best response to $\mathbf{q}$ if it is generic, and if

$$\pi^{\varepsilon}(\mathbf{p},\mathbf{q}) \geq \pi^{\varepsilon}(\mathbf{p}',\mathbf{q}) \quad \text{for all generic strategies } \mathbf{p}'. \tag{30}$$

Then we can again show that one can always find a generic best response to a memory-1 strategy among the deterministic reactive strategies.

Proposition 5 (Optimality of deterministic reactive strategies in games with errors)**.**
Let $\mathbf{q}$ *be some given memory-1 strategy and let* $\mathbf{p}\in[0,1]^2$ *be an arbitrary reactive strategy. Then, there is a deterministic reactive strategy* $\mathbf{p}'\in\{0,1\}^2$ *for which* $\pi^{\varepsilon}(\mathbf{p}',\mathbf{q}) \geq \pi^{\varepsilon}(\mathbf{p},\mathbf{q})$.

Similar to before, we say a memory-1 strategy $\mathbf{q}$ is a Nash equilibrium if $\pi^{\varepsilon}(\mathbf{q},\mathbf{q}) \geq \pi^{\varepsilon}(\mathbf{q}',\mathbf{q})$ for any generic deviation strategy $\mathbf{q}'$. **Proposition 5** then again allows us to identify memory-1 Nash equilibria more effectively. It suffices to compare the payoff of the strategy $\mathbf{q}$ against itself to the payoffs one could achieve with deterministic reactive strategies. However, now the payoff expressions are more complex. Using Eq. (26), we calculate the self-payoff as

$$\pi^{\varepsilon}(\mathbf{q},\mathbf{q}) = \frac{(1-q^{\varepsilon}_{CC}+q^{\varepsilon}_{DC})q^{\varepsilon}_{DD}}{(1-q^{\varepsilon}_{CC})(1-q^{\varepsilon}_{CD}+q^{\varepsilon}_{DD})+(1-q^{\varepsilon}_{CC}+q^{\varepsilon}_{DC})q^{\varepsilon}_{DD}} \cdot (b-c). \tag{31}$$

For the possible deviations towards deterministic reactive strategies, we obtain

$$\begin{aligned}
\pi^{\varepsilon}(ALLD,\mathbf{q}) &= \frac{\varepsilon q^{\varepsilon}_{DC} + (1-\varepsilon)q^{\varepsilon}_{DD}}{1-\varepsilon(q^{\varepsilon}_{CC}-q^{\varepsilon}_{DC})-(1-\varepsilon)\,(q^{\varepsilon}_{CD}-q^{\varepsilon}_{DD})} \cdot b - \varepsilon \cdot c \\[2ex]
\pi^{\varepsilon}(ATFT,\mathbf{q}) &= \frac{(1-\varepsilon)q^{\varepsilon}_{DC} + \varepsilon q^{\varepsilon}_{DD}}{1-\varepsilon(q^{\varepsilon}_{CC}-q^{\varepsilon}_{DD})-(1-\varepsilon)\,(q^{\varepsilon}_{CD}-q^{\varepsilon}_{DC})} \cdot b \\
&\quad - \frac{1-\varepsilon-\varepsilon(1-\varepsilon)q^{\varepsilon}_{CC}-(1-\varepsilon)^2q^{\varepsilon}_{CD}+\varepsilon(1-\varepsilon)q^{\varepsilon}_{DC}+\varepsilon^2q^{\varepsilon}_{DD}}{1-\varepsilon(q^{\varepsilon}_{CC}-q^{\varepsilon}_{DD})-(1-\varepsilon)\,(q^{\varepsilon}_{CD}-q^{\varepsilon}_{DC})} \cdot c \\[2ex]
\pi^{\varepsilon}(TFT,\mathbf{q}) &= \frac{\varepsilon q^{\varepsilon}_{DC} + (1-\varepsilon)q^{\varepsilon}_{DD}}{1-(1-\varepsilon)(q^{\varepsilon}_{CC}-q^{\varepsilon}_{DD})-\varepsilon(q^{\varepsilon}_{CD}-q^{\varepsilon}_{DC})} \cdot b \\
&\quad - \frac{\varepsilon-\varepsilon(1-\varepsilon)q^{\varepsilon}_{CC}-\varepsilon^2q^{\varepsilon}_{CD}+\varepsilon(1-\varepsilon)q^{\varepsilon}_{DC}+(1-\varepsilon)^2q^{\varepsilon}_{DD}}{1-(1-\varepsilon)(q^{\varepsilon}_{CC}-q^{\varepsilon}_{DD})-\varepsilon(q^{\varepsilon}_{CD}-q^{\varepsilon}_{DC})} \cdot c \\[2ex]
\pi^{\varepsilon}(ALLC,\mathbf{q}) &= \frac{(1-\varepsilon)q^{\varepsilon}_{DC}+\varepsilon q^{\varepsilon}_{DD}}{1-(1-\varepsilon)(q^{\varepsilon}_{CC}-q^{\varepsilon}_{DC})-\varepsilon(q^{\varepsilon}_{CD}-q^{\varepsilon}_{DD})} \cdot b - (1-\varepsilon)\cdot c
\end{aligned} \tag{32}$$

We define partners, defectors, and equalizers analogously to the case without errors. We call a memory-1 strategy $\mathbf{q}$ self-cooperative if it yields the mutual cooperation payoff against itself as errors become rare, $\varepsilon \to 0$ (in particular, it must satisfy $q_{CC} = 1$). Similarly, $\mathbf{q}$ is self-defective if it yields the self-defection payoff against itself in the limit of rare errors (in particular, $q_{DD}=0$). Partners are again those

Nash equilibria that are self-cooperative, and defectors as those Nash equilibria that are self-defective. As before, a strategy is an equalizer for a given error probability ε if any generic co-player yields the same payoff against that strategy. The following then generalizes the results of **Theorems 1** and **2** to the case of alternating games with errors.

Theorem 3 (Classification of Nash equilibria in alternating games with errors)**.**
Consider a memory-1 strategy $\mathbf{q}=(q_{CC}, q_{CD}, q_{DC}, q_{DD})$ *for an alternating game with error probability* $0<\varepsilon<\frac{1}{2}\left(1-\frac{c}{b}\right)$. *Then, the following are equivalent.*

1. *The strategy* $\mathbf{q}$ *is a generic Nash equilibrium.*
2. *The strategy* $\mathbf{q}$ *satisfies*

$$\pi^{\varepsilon}(\mathbf{q},\mathbf{q}) \geq \max\left(\pi^{\varepsilon}(\text{ALLD},\mathbf{q}),\ \pi^{\varepsilon}(\text{ATFT},\mathbf{q}),\ \pi^{\varepsilon}(\text{TFT},\mathbf{q}),\ \pi^{\varepsilon}(\text{ALLC},\mathbf{q})\ \right). \tag{33}$$

3. *The strategy* $\mathbf{q}$ *is either a partner, a defector, or an equalizer. It is a partner if and only If*

$$\begin{aligned} q_{CC} &= q_{DC} = 1, \\ q_{CD} &\leq 1-\frac{c}{(1-2\varepsilon)b}, \\ q_{DD} &= \frac{(1-2\varepsilon)\,(b+\varepsilon c\, q_{CD}) - c}{(1-2\varepsilon)\,(b+\varepsilon c)}. \end{aligned} \tag{34}$$

   It is a defector if and only if it is either ALLD *or*

$$\begin{aligned} q_{DD} &= q_{CD} = 0, \\ q_{DC} &\leq \frac{c}{(1-2\varepsilon)b}, \\ q_{CC} &= \frac{\varepsilon(1-2\varepsilon)c\, q_{DC} + c}{(1-2\varepsilon)(b+\varepsilon c)}. \end{aligned} \tag{35}$$

   It is an equalizer if and only if

$$\begin{aligned} q_{CD} &= \frac{(1-2\varepsilon)(b\, q_{CC} - c\, q_{DD}) - c}{(1-2\varepsilon)\,(b-c)} \\ q_{DC} &= \frac{(1-2\varepsilon)(b\, q_{DD} - c\, q_{CC}) + c}{(1-2\varepsilon)\,(b-c)}. \end{aligned} \tag{36}$$

Several remarks are in order:

(a) Dimension of the Nash equilibrium classes. Errors lead to a discontinuous reduction in the number of Nash equilibria. For example, for any arbitrarily small (but positive) error probability the set of partners is now one-dimensional instead of three-dimensional. The proof of **Theorem 3** suggests there are two reasons for this reduction.

First, in games without errors, $p_{CC} = 1$ is sufficient to ensure that the reactive marginalization of $\mathbf{q}$ with respect to itself satisfies $\tilde{q}_C = 1$, see Eq. (10). Game outcomes different from mutual cooperation are (almost) never visited, and hence $v_{DC} = 0$. For games with errors, this is no longer true. Independent of the players' strategies, each game outcome occurs at least when both players choose the respective action by mistake, such that $v_{a\tilde{a}} \geq \varepsilon^2$ for all $(a, \tilde{a}) \in \{CC, CD, DC, DD\}$. To guarantee that the reactive marginalization still satisfies $\tilde{q}_C = 1$, we therefore additionally need to require that $q_{DC} = 1$.

Second, in alternating games without errors, a partner $\mathbf{q}$ cannot be invaded by the two boundary strategies *ALLC* and *TFT*. Instead, all three strategies yield the same payoff $b - c$ against $\mathbf{q}$. In contrast, in games with errors, the payoffs of the three strategies are generally different. Moreover, by **Lemma 1** the payoff $\pi^\varepsilon(\mathbf{q}, \mathbf{q})$ is either strictly in between $\pi^\varepsilon(\mathit{TFT}, \mathbf{q})$ and $\pi^\varepsilon(\mathit{ALLC}, \mathbf{q})$, or all three payoffs are the same. For $\mathbf{q}$ to be a Nash equilibrium, we thus need to require

$$\pi^\varepsilon(\mathit{TFT}, \mathbf{q}) = \pi^\varepsilon(\mathbf{q}, \mathbf{q}) = \pi^\varepsilon(\mathit{ALLC}, \mathbf{q}). \tag{37}$$

This equality is equivalent to the last equality in condition (34). Finally, the upper bound on q_{CD} ensures that neither a deviation to $ALLD$ nor to *ATFT* is profitable.

As a consequence of these observations, we conclude that many of the well-known self-cooperative memory-1 strategies fail to be partners in alternating games with errors. In particular, *TFT*, *WSLS*, *GRIM* and *FBF* are either no longer self-cooperative, or they are no longer Nash equilibria. Similar considerations also explain why the class of defectors is now one-dimensional.

(b) Evolutionary stability. While all partner strategies for the alternating game are Nash equilibria by definition, we note that none of them are evolutionarily stable in the sense of Maynard-Smith[17]. Instead, by (37), mutants who either adopt *TFT* or *ALLC* can invade through neutral drift – in fact, due to the monotonicity property in **Lemma 1**, *any* mutant strategy $\mathbf{q}'$ with $q_{CC} = q_{DC} = 1$ yields the same payoff against $\mathbf{q}$ as $\mathbf{q}$ does against itself.

Thus, as a corollary of **Theorem 3**, we conclude that in alternating games with errors, there are no evolutionarily stable memory-1 strategies that sustain cooperation. Note that this result is different from previous work suggesting that no strategy in the simultaneous game is stable[18–21]. This previous work considers games without errors. Only in that case does it show that for each Nash equilibrium one can identify neutral mutant strategies that eventually lead out of that equilibrium. These arguments do not apply to games with errors, where evolutionary stability is generally feasible[22]. For example, for games with $b > 2c$, one can show that *WSLS* is evolutionarily stable in

the simultaneous game, provided the error probability is positive but sufficiently small[1,23].

(c) Existence of the Nash equilibrium classes and comparative statics. Partners do not exist for all parameter values. Because q_{CD} and q_{DD} need to be values in the unit interval, condition (34) implies that partners exist if and only if

$$\varepsilon < \frac{1}{2}\left(1 - \frac{c}{b}\right). \tag{38}$$

In particular, the conditions for partners to exist are easiest to satisfy if either implementation errors are sufficiently rare, or if the benefit of cooperation is large compared to its costs. The same condition (38) also determines whether or not equalizer strategies exist.

In contrast, defectors exist for all parameter values $b > c$ and $0 \leq \varepsilon \leq 1/2$, because *ALLD* is always a Nash equilibrium. When condition (38) does not hold, mutual defection is in fact the only behavior that can be sustained in equilibrium.

(d) Examples of partner strategies. Provided condition (38) holds, the set of partners is given by the line segment connecting the two strategies

$$\mathbf{q}' = \left(1,\ 0,\ 1,\ \frac{(1-2\varepsilon)b-c}{(1-2\varepsilon)(b+\varepsilon c)}\right) \tag{39}$$

$$\mathbf{q}'' = \left(1,\ \frac{(1-2\varepsilon)b-c}{(1-2\varepsilon)b},\ 1,\ \frac{(1-2\varepsilon)b-c}{(1-2\varepsilon)b}\right) \tag{40}$$

In particular, we note that while q_{CD} may be chosen to be zero, q_{DD} always needs to be strictly in between zero and one. The first example above, $\mathbf{q}'$ can be considered as a stochastic version of Firm-but-Fair, and hence we refer to it as *SFBF*. Strategies resembling *SFBF* have been described previously. For example, Nowak and Sigmund[2] observe that the simulations in their Figure 3 converge to the strategy $(1, 0, 1, 2/3)$. Using their parameters $b = 3$, $c = 1$ and $\varepsilon = 0.001$, this is exactly what is predicted by expression (39). The second example above, $\mathbf{q}''$ corresponds to the well-known Generous Tit-for-Tat strategy[24,25] (*GTFT*) which has been previously described for the simultaneous game. According to Eqs. (39) and (40), *GTFT* is the only partner among the reactive strategies. For reactive strategies, the simultaneous and the alternating game coincide with respect to their dynamics[2]. In this light, the fact that *GTFT* also makes an appearance in the alternating game is somewhat less surprising.

In the limit of rare errors, $\varepsilon \to 0$, the above expressions simplify further. We obtain

$$\mathbf{q}' = \left(1,\ 0,\ 1,\ 1-\frac{c}{b}\right) \quad \text{and} \quad \mathbf{q}'' = \left(1,\ 1-\frac{c}{b},\ 1,\ 1-\frac{c}{b}\right) \tag{41}$$

(e) Alternative specification of errors. Throughout this section we have assumed that errors originate from players who misimplement their intended actions with a constant probability ε. In this case,

a player's effective strategy $\mathbf{q}^{\varepsilon}$ is a linear function of the player's actual strategy $\mathbf{q}$, as described by Eq. (23). However, analogous results can be derived for more general error mappings. Our main results only require that the effect of errors on a player's strategy can be described by a strictly monotonic and bijective transformation $\varphi^{\varepsilon} : [0,1]^4 \to [\varepsilon, 1-\varepsilon]^4$ and that *ALLD*, *ATFT*, *TFT*, *ALLC* are mapped to the values in Eq. (25). Under that more general assumption, condition (33) continues to characterize which memory-1 strategies $\mathbf{q}$ are equilibria. Only the exact description of partners, defectors, and equalizers needs to be adapted correspondingly. In addition, one can also extend our results to cases where the error rate depends on the previous outcome, or where it depends on the player's intended action. In this way, one could model cases in which a player who intends to cooperate is more likely to make a mistake than a player who intends to defect.

Supplementary Note 3: Extensions of the baseline model

The baseline model makes a number of simplifying assumptions: (*i*) the game is infinitely repeated; (*ii*) the players move in a strictly alternating fashion; (*iii*) the simulations only take into account memory-1 strategies; (*iv*) interactions take place in a well-mixed population; and (*v*) mutations are global. In the following, we study the impact of each of these assumptions in more detail. In each case, we explore how the respective assumption affects emerging cooperation rates and the strategies that evolve.

Finitely repeated games

Motivation. Our baseline model considers an infinitely repeated game with no discounting of the future. There are two major reasons why the analysis of such games is useful. First, from a mathematical perspective, infinitely repeated games are more convenient to work with because their results tend to be independent of the players' behavior in the early rounds of the game. This in turn allows researchers to consider a simpler strategy space; the players' first-round behavior no longer needs to be specified[8]. Second, such games often serve as a good approximation for games where the number of rounds is large but finite[26]. In many cases, results for finitely repeated games resemble the results of infinitely repeated games already for a moderate number of rounds[27]. To elaborate on the above two points, and to extend the results of the baseline model, in the following we study a model in which players only engage in finitely many interactions.

Game setup. We assume the game proceeds in rounds. In the simultaneous game, the two players move simultaneously in each round. In the alternating game, one player moves first and the other player moves second. Here, the player who moves first is determined randomly, but kept constant during the game. After each round, the game continues for another round with a constant continuation probability δ. As a

result, the expected number of rounds follows a geometric distribution with mean $1/(1-\delta)$. Let $v_{a_1,a_2}(t)$ denote the conditional probability that the two players choose actions a_1 and a_2 in round t, given that round t is reached. Then we can calculate the average probability to observe the respective outcome over the course of the entire game as

$$v_{a_1,a_2} := (1-\delta)\sum_{t=0}^{\infty}\delta^t \cdot v_{a_1,a_2}(t). \tag{42}$$

In the limiting case that there is always another round, $\delta \to 1$, this weighted average converges to the time average (1) of the baseline model, provided the limit in Eq. (1) exists. As before, we collect these four averages in a vector $\mathbf{v} = (v_{CC}, v_{CD}, v_{DC}, v_{DD})$. Based on this vector, we can compute the players' payoffs in the same way as in the baseline model, using Eq. (3).

Memory-1 strategies. To introduce memory-1 strategies for finitely repeated games, we distinguish between the simultaneous and the alternating game. In the simultaneous game, memory-1 strategies take the form[11]

$$\mathbf{p} = (p_{00};\ p_{CC}, p_{CD}, p_{DC}, p_{DD}). \tag{43}$$

The first entry p_{00} is the player's probability to cooperate in the initial round. The other entries p_{ij} are the respective conditional cooperation probabilities in all subsequent rounds, as defined in the baseline model. In the alternating game, memory-1 strategies take the form

$$\mathbf{p} = (p_{00};\ p_{0C}, p_{0D};\ p_{CC}, p_{CD}, p_{DC}, p_{DD}). \tag{44}$$

Here, p_{00} is the probability to cooperate in the first round if the focal player moves first. The next two probabilities p_{0C} and p_{0D} are the player's probability to cooperate in the first round if the player moves second. In that case, the focal player may condition its decision on the co-player's first round behavior (C or D). The other entries p_{ij} are again the conditional cooperation probabilities that the focal player applies in all subsequent rounds. In particular, we note that while strategies in the baseline model are 4-dimensional, they are now 5-dimensional in the case of simultaneous games, and 7-dimensional in the case of alternating games.

Explicit formulas for the players' payoffs. When two memory-1 players interact, their payoffs can be computed in a similar way as in the baseline model[26]. To this end, we consider first the simultaneous game. Suppose the strategies of the two players are

$$\begin{aligned}\mathbf{p} &= (p_{00};\ p_{CC}, p_{CD}, p_{DC}, p_{DD})\\ \mathbf{q} &= (q_{00};\ q_{CC}, q_{CD}, q_{DC}, q_{DD}),\end{aligned} \tag{45}$$

respectively. Then the outcome distribution in the initial round is

$$\begin{aligned} \mathbf{v}_0 &:= \big(v_{CC}(0),\ v_{CD}(0),\ v_{DC}(0),\ v_{DD}(0)\big) \\ &= \Big(p_{00}\!\cdot\! q_{00},\ p_{00}\!\cdot\!(1\!-\!q_{00}),\ (1\!-\!p_{00})\!\cdot\! q_{00},\ (1\!-\!p_{00})\!\cdot\!(1\!-\!q_{00})\Big). \end{aligned} \tag{46}$$

Given this initial outcome distribution, we can iteratively compute all subsequent distributions as

$$\mathbf{v}(t) = \mathbf{v}_0 \cdot M_S^t. \tag{47}$$

Here, $M_S = M_S(\mathbf{p}, \mathbf{q})$ is the standard transition matrix for the simultaneous game, as defined by Eq. (8). For the average distribution $\mathbf{v}$ according to Eq. (42), we therefore obtain

$$\mathbf{v} = (1\!-\!\delta)\sum_{t=0}^{\infty}\delta^t \cdot \mathbf{v}(t) = (1\!-\!\delta)\mathbf{v}_0\sum_{t=0}^{\infty}(\delta M_S)^t = (1\!-\!\delta)\mathbf{v}_0(I - \delta M_S)^{-1}. \tag{48}$$

Here, I denotes the 4×4 identity matrix, and $(I - \delta M_S)^{-1}$ refers to the respective inverse matrix. Based on $\mathbf{v}$, we compute player's payoffs using Eq. (3). That is,

$$\begin{aligned} \pi_1 &= b\!\cdot\!(v_{CC}\!+\!v_{DC}) - c\!\cdot\!(v_{CC}\!+\!v_{CD}) \\ \pi_2 &= b\!\cdot\!(v_{CC}\!+\!v_{CD}) - c\!\cdot\!(v_{CC}\!+\!v_{DC}). \end{aligned} \tag{49}$$

The payoffs of the alternating game can be computed analogously. However, here we have to distinguish two cases, depending on which of the two players moves first. Suppose the two players' strategies are given by

$$\begin{aligned} \mathbf{p} &= (p_{00};\ p_{0C}, p_{0D};\ p_{CC}, p_{CD}, p_{DC}, p_{DD}) \\ \mathbf{q} &= (q_{00};\ q_{0C}, q_{0D};\ q_{CC}, q_{CD}, q_{DC}, q_{DD}). \end{aligned} \tag{50}$$

If it is player 1 who moves first, the initial outcome distribution is

$$\mathbf{v}_0^{(1)} = \Big(p_{00}\!\cdot\! q_{0C},\ p_{00}\!\cdot\!(1\!-\!q_{0C}),\ (1\!-\!p_{00})\!\cdot\! q_{0D},\ (1\!-\!p_{00})\!\cdot\!(1\!-\!q_{0D})\Big). \tag{51}$$

The respective average distribution over the entire game can then be calculated as

$$\mathbf{v}^{(1)} = (1\!-\!\delta)\mathbf{v}_0^{(1)}\big(I - \delta M_A(\mathbf{p},\mathbf{q})\big)^{-1}, \tag{52}$$

where $M_A(\mathbf{p}, \mathbf{q})$ is the standard transition matrix (5) of the alternating game. Similarly, if it is player 2 who moves first, the initial distribution is

$$\mathbf{v}_0^{(2)} = \Big(q_{00}\!\cdot\! p_{0C},\ q_{00}\!\cdot\!(1\!-\!p_{0C}),\ (1\!-\!q_{00})\!\cdot\! p_{0D},\ (1\!-\!q_{00})\!\cdot\!(1\!-\!p_{0D})\Big). \tag{53}$$

The average distribution becomes

$$\mathbf{v}^{(2)} = (1-\delta)\mathbf{v}_0^{(2)}\big(I - \delta M_A(\mathbf{q},\mathbf{p})\big)^{-1}. \tag{54}$$

Because each of the two players is equally likely to move first, average payoffs are now

$$\begin{aligned}\pi_1 &= b\cdot\Big(\tfrac{1}{2}\big(v_{CC}^{(1)}+v_{DC}^{(1)}\big) + \tfrac{1}{2}\big(v_{CC}^{(2)}+v_{CD}^{(2)}\big)\Big) - c\cdot\Big(\tfrac{1}{2}\big(v_{CC}^{(1)}+v_{CD}^{(1)}\big) + \tfrac{1}{2}\big(v_{CC}^{(2)}+v_{DC}^{(2)}\big)\Big)\\ \pi_2 &= b\cdot\Big(\tfrac{1}{2}\big(v_{CC}^{(1)}+v_{CD}^{(1)}\big) + \tfrac{1}{2}\big(v_{CC}^{(2)}+v_{DC}^{(2)}\big)\Big) - c\cdot\Big(\tfrac{1}{2}\big(v_{CC}^{(1)}+v_{DC}^{(1)}\big) + \tfrac{1}{2}\big(v_{CC}^{(2)}+v_{CD}^{(2)}\big)\Big).\end{aligned} \tag{55}$$

Evolutionary dynamics. Given the payoffs (49) and (55), we can explore the evolutionary dynamics of the finitely repeated game with the same process we used for the infinitely repeated game. That is, again we consider a finite and well-mixed population in which players adopt new strategies by imitation and mutation, as described in the main text.

Fig. 6a,b shows the corresponding results for a continuation probability of $\delta = 0.96$, such that individuals interact on average for 25 rounds. The figure suggests that the main evolutionary findings are in qualitative agreement with the findings of the baseline model. First, and as in the baseline model, the simultaneous game is slightly more conducive to the evolution of cooperation compared to the alternating game (**Fig. 6a**). Second, the self-cooperative strategies that evolve in the simultaneous game are markedly different from the self-cooperative strategies that evolve in the alternating game (**Fig. 6b**). In the simultaneous game, the average self-cooperative strategy shares the main characteristics of win-stay lose-shift[28]. Players are most likely to cooperate after mutual cooperation or mutual defection. In the alternating game, evolving strategies rather resemble a mixture of Generous Tit-for-Tat and Stochastic Firm-but-Fair. Here, players are most likely to cooperate if the co-player cooperated in the previous round. In addition, players exhibit a positive probability to cooperate after both players defected. In either game, the payoff derived in the first round only has a modest impact on the player's overall fitness, given the game length. As a result, the values of q_{00}, q_{0C}, q_{0D} are close to 1/2, as one would expect from traits that are almost neutral.

We explore the dynamics for other continuation probabilities in **Fig. 6c** and **Supplementary Fig. 5**. If the continuation probability exceeds some moderate threshold, $\delta \approx 0.8$ (which corresponds to games with five rounds in expectation), the qualitative results are largely comparable to the results of the baseline model. Below this threshold, cooperation is rare in both the simultaneous and the alternating game.

Discussion of the model. We note that by structuring the game into rounds, we implicitly assume that the two players always make the same number of decisions in the alternating game, independent of the realized length of the game. In particular, every time the first player makes a decision, this player can be sure that the second player will have an opportunity to reciprocate. We have made this assumption to make it easier to compare the alternating game to the simultaneous game. By making sure that both

players make the same number of decisions, the continuation probability δ has an analogous interpretation in both games. Alternatively, we could have assumed that after each player's decision, the game stops with a certain probability. That alternative scenario is a special case of the model that we consider in the following.

Irregular alternation patterns

Motivation. For both the finitely and the infinitely repeated alternating game, we assumed in the baseline model that players move in a strictly alternating fashion. That is, every time player 1 makes a decision, it is player 2 who makes the next decision. Conversely, every time, player 2 makes a decision, it is player 1 who makes the next decision (provided there is another round). Instead, here we explore what happens when the alternation pattern can be more irregular, such that players may have to make a decision two times in a row before it's the other player's turn to move.

Game setup. To allow for such irregular patterns, we structure the alternating game into a sequence of moves. The player who makes the initial move is determined randomly, with each player having the same chance to move first. After a player has made a move (by deciding whether or not to cooperate), there is a constant probability λ that the game continues. If the game continues, the player who makes the next move is determined randomly. With probability s (the 'switching probability'), it is the other player who makes the next move. With the converse probability $\bar{s} := 1-s$, it is the same player. In the special case $s=1$, we recover a setup in which the two players move in a strictly alternating fashion. If $s=1/2$, the next move is assigned completely randomly, independent of who moved before. Finally, if $s=0$, there is no alternation at all; the player who moves first is guaranteed to move in all subsequent interactions. The payoffs of each player are defined analogously to the previous cases, by averaging the received benefits and paid costs over all moves of the two players (for more detail, see further below).

Memory-1 strategies. Similar to the baseline model, we assume that players condition their behavior only on the respective last move of either player (that is, they remember one move per player). In the case of irregular interaction patterns, such strategies take the form of a nine-dimensional vector,

$$\mathbf{p} = (p_{00};\ p_{C0}, p_{D0};\ p_{0C}, p_{0D};\ p_{CC}, p_{CD}, p_{DC}, p_{DD}). \tag{56}$$

Here, p_{00} is the player's cooperation probability if no player has moved before. The next two entries, p_{C0} and p_{D0} are the player's cooperation probability if only the focal player has moved before (but not the co-player). The other two entries, p_{0C} and p_{0D} are the player's cooperation probability if only the co-player has moved before (but not the focal player). And finally, the entries p_{ij} with $i, j \in \{C, D\}$ are the usual conditional cooperation probabilities in all subsequent rounds.

Explicit formulas for the players' payoffs. Again assuming that both players use memory-1 strategies,

we can compute their payoffs explicitly. To this end, suppose the strategies of player 1 and player 2 are

$$\begin{aligned}\mathbf{p} &= (p_{00};\ p_{C0}, p_{D0};\ p_{0C}, p_{0D};\ p_{CC}, p_{CD}, p_{DC}, p_{DD})\\ \mathbf{q} &= (q_{00};\ q_{C0}, q_{D0};\ q_{0C}, q_{0D};\ q_{CC}, q_{CD}, q_{DC}, q_{DD})\end{aligned} \tag{57}$$

respectively. We describe the dynamics among the two players by a Markov chain with twelve possible states. The twelve states are (in this order):

$$\begin{aligned}&(1,C,\emptyset),\ \ (1,D,\emptyset),\ \ (2,\emptyset,C),\ \ (2,\emptyset,D),\\ &(1,C,C),\ (1,C,D),\ (1,D,C),\ (1,D,D),\ (2,C,C),\ (2,C,D),\ (2,D,C),\ (2,D,D).\end{aligned} \tag{58}$$

Here, the state (i, a_1, a_2) refers to the case that the previous move was made by player $i \in \{1,2\}$, and that after this move, the last move by either player is $a_1, a_2 \in \{C, D, \emptyset\}$; the empty set symbol indicates that the respective player did not make a move yet. We obtain the following distribution for the state of the Markov chain after the first move:

$$\mathbf{v}_0 = \Big(\frac{p_{00}}{2}, \frac{\bar{p}_{00}}{2}, \frac{q_{00}}{2}, \frac{\bar{q}_{00}}{2}, 0,\ 0,\ 0,\ 0,\ 0,\ 0,\ 0,\ 0\Big). \tag{59}$$

For this initial distribution, we have used the shortcut notation $\bar{p}_{00} := 1-p_{00}$ and $\bar{q}_{00} := 1-q_{00}$. The transition matrix of the Markov chain is given by

$$M_I(\mathbf{p},\mathbf{q}) = \begin{pmatrix}
\bar{s}p_{C0} & \bar{s}\bar{p}_{C0} & 0 & 0 & 0 & 0 & 0 & 0 & sq_{0C} & s\bar{q}_{0C} & 0 & 0\\
\bar{s}p_{D0} & \bar{s}\bar{p}_{D0} & 0 & 0 & 0 & 0 & 0 & 0 & 0 & 0 & sq_{0D} & s\bar{q}_{0D}\\
0 & 0 & \bar{s}q_{C0} & \bar{s}\bar{q}_{C0} & sp_{0C} & 0 & s\bar{p}_{0C} & 0 & 0 & 0 & 0 & 0\\
0 & 0 & \bar{s}q_{D0} & \bar{s}\bar{q}_{D0} & 0 & sp_{0D} & 0 & s\bar{p}_{0D} & 0 & 0 & 0 & 0\\
0 & 0 & 0 & 0 & \bar{s}p_{CC} & 0 & \bar{s}\bar{p}_{CC} & 0 & sq_{CC} & s\bar{q}_{CC} & 0 & 0\\
0 & 0 & 0 & 0 & 0 & \bar{s}p_{CD} & 0 & \bar{s}\bar{p}_{CD} & sq_{DC} & s\bar{q}_{DC} & 0 & 0\\
0 & 0 & 0 & 0 & \bar{s}p_{DC} & 0 & \bar{s}\bar{p}_{DC} & 0 & 0 & 0 & sq_{CD} & s\bar{q}_{CD}\\
0 & 0 & 0 & 0 & 0 & \bar{s}p_{DD} & 0 & \bar{s}\bar{p}_{DD} & 0 & 0 & sq_{DD} & s\bar{q}_{DD}\\
0 & 0 & 0 & 0 & sp_{CC} & 0 & s\bar{p}_{CC} & 0 & \bar{s}q_{CC} & \bar{s}\bar{q}_{CC} & 0 & 0\\
0 & 0 & 0 & 0 & 0 & sp_{CD} & 0 & s\bar{p}_{CD} & \bar{s}q_{DC} & \bar{s}\bar{q}_{DC} & 0 & 0\\
0 & 0 & 0 & 0 & sp_{DC} & 0 & s\bar{p}_{DC} & 0 & 0 & 0 & \bar{s}q_{CD} & \bar{s}\bar{q}_{CD}\\
0 & 0 & 0 & 0 & 0 & sp_{DD} & 0 & s\bar{p}_{DD} & 0 & 0 & \bar{s}q_{DD} & \bar{s}\bar{q}_{DD}
\end{pmatrix}. \tag{60}$$

Based on the initial distribution $\mathbf{v}_0$ and on the transition matrix $M_I(\mathbf{p},\mathbf{q})$, we can again compute the average distribution to observe any of the twelve states over the course of the game as

$$\mathbf{v} = (1-\lambda)\mathbf{v}_0\big(I - \lambda M_I(\mathbf{p},\mathbf{q})\big)^{-1}. \tag{61}$$

The payoffs of the two players are then given by the formula

$$\begin{aligned}\pi_1 &= 2\,\mathbf{v}\cdot(-c, 0, b, 0, -c, -c, 0, 0, b, 0, b, 0)^\top\\ \pi_2 &= 2\,\mathbf{v}\cdot(b, 0, -c, 0, b, b, 0, 0, -c, 0, -c, 0)^\top.\end{aligned} \tag{62}$$

Here, the factor of two is a normalization constant to take into account that in half of the rounds it's the focal player who moves, whereas in the other half it's the co-player. This constant ensures that two unconditional cooperators obtain an expected payoff of $(1-\varepsilon)(b-c)$, as one would expect.

Evolutionary dynamics. We explore the evolutionary dynamics of the game with irregular alternation patterns using the same process we used for the baseline model. In **Fig. 6d** we compare two scenarios. In the first scenario, we assume that players strictly alternate ($s=1$). In the other scenario, it is randomly determined which player moves next ($s=1/2$). In both cases, we consider the dynamics of a game that is infinitely repeated ($\lambda\to1$), with a moderate error rate ($\varepsilon=0.02$) and rare mutations ($\mu\to1$). We observe that a strictly alternating game leads to higher cooperation rates. To explore this result in more detail, we also recorded which self-cooperative strategies the players are most likely to adopt over the course of time. Surprisingly, both the strictly alternating and the randomly alternating game lead to overall similar strategies (**Fig. 6e**). In each case, the average strategy reflects the basic patterns of Stochastic Firm-but-Fair. However, in the strictly alternating case, these self-cooperative strategies tend to be more robust. When players are strictly alternating, it takes on average 1,600 mutants until a resident self-cooperative strategy is successfully invaded. In contrast, for randomly alternating games, this number drops to 980 mutants. More generally, we observe that cooperation is most likely to evolve the more likely players alternate in a regular manner (**Fig. 6f**). In the extreme case that players do not alternate at all ($s=0$), cooperation does not evolve, as one may expect.

In addition to these simulation results for infinitely repeated games, we have also explored the dynamics when the number of rounds is finite (**Supplementary Fig. 6**). There we show the outcome of two sets of simulations, one in the absence of errors ($\varepsilon=0$) and one with a moderate error rate ($\varepsilon=0.02$). In both cases, we vary the expected number of rounds (between 1 and 100), and the switching probability (between 0 and 1). These simulations confirm the previously observed regularities for alternating games: Cooperation is most likely to evolve when (*i*) errors are rare, (*ii*) players interact for many rounds, and (*iii*) players alternate in a regular fashion.

Memory-2 strategies

Motivation. For all evolutionary results so far, we have assumed that players only take into account each player's last action. While it has been argued that memory-1 strategies are good approximations for human behavior in laboratory experiments in simultaneous games[29], it is natural to ask which of our qualitative results depend on the one-round memory assumption. Exploring the evolutionary dynamics among more complex strategies is not straightforward because the number of available strategies increases super-exponentially in the players' memory capacity. In the baseline case of an infinitely repeated game, there are 16 pure memory-1 strategies, 65,536 pure memory-2 strategies, and $1.84\cdot10^{19}$ pure memory-3 strategies[1]. In the following, we thus confine ourselves to pure memory-2 strategies. Those are all strategies that consider the last two moves of each player, and for which the corresponding

cooperation probability (in the absence of errors) is either zero or one.

Memory-2 strategies. For simplicity, we shall only consider the case of infinitely repeated games here (although the case of finitely games can be treated similarly, as discussed in the previous sections). In infinitely repeated games, memory-2 strategies can be represented by a 16-dimensional vector,

$$\mathbf{p} = \Big(p_{\substack{CC\\CC}}, p_{\substack{CC\\CD}}, p_{\substack{CD\\CC}}, p_{\substack{CD\\CD}}, p_{\substack{CC\\DC}}, p_{\substack{CC\\DD}}, p_{\substack{CD\\DC}}, p_{\substack{CD\\DD}}, p_{\substack{DC\\CC}}, p_{\substack{DC\\CD}}, p_{\substack{DD\\CC}}, p_{\substack{DD\\CD}}, p_{\substack{DC\\DC}}, p_{\substack{DC\\DD}}, p_{\substack{DD\\DC}}, p_{\substack{DD\\DD}} \Big). \tag{63}$$

The entries again reflect the player's conditional cooperation probabilities. The upper two indices of an entry represent the last two moves of the focal player (with the very last move coming first and the second-to last move coming second). Analogously, the lower two indices represent the last two moves of the co-player. The space of memory-2 strategies trivially contains the set of all memory-1 strategies as a subset. For example, within the space of memory-2 strategies, Tit-for-Tat takes the form

$$\mathbf{p} = (1, 1, 1, 1, 0, 0, 0, 0, 1, 1, 1, 1, 0, 0, 0, 0). \tag{64}$$

Because each pure memory-2 strategy is a 16-dimensional vector, and each entry is either zero or one, there are indeed $2^{16} = 65,536$ such strategies in total.

Explicit formulas for the players' payoffs. The payoffs of two memory-2 players can again be computed with a Markov chain approach. To this end, suppose the strategies of player 1 and player 2 are $\mathbf{p}$ and $\mathbf{q}$, respectively, and each of these strategies is of the form (63). Then, the respective Markov chain has sixteen possible states, summarizing the last two moves of either player, $\substack{CC\\CC}, \substack{CC\\CD}, \substack{CD\\CC}, \ldots, \substack{DD\\DD}$. Slightly abusing notation, here the upper indices refer to the past two actions of player 1 and the lower two indices refer to the past two actions of player 2. Given this ordering of the states, the transition matrix M_A of the alternating game takes the following form (The transition matrix for the simultaneous game takes a similar form, and has been derived elsewhere[1]):

$$
\begin{pmatrix}
p_{\substack{CC\\CC}}q_{\substack{CC\\CC}} & 0 & 0 & 0 & p_{\substack{CC\\CC}}\bar{q}_{\substack{CC\\CC}} & 0 & 0 & 0 & \bar{p}_{\substack{CC\\CC}}q_{\substack{CC\\DC}} & 0 & 0 & 0 & \bar{p}_{\substack{CC\\CC}}\bar{q}_{\substack{CC\\DC}} & 0 & 0 & 0 \\
p_{\substack{CC\\CD}}q_{\substack{CD\\CC}} & 0 & 0 & 0 & p_{\substack{CC\\CD}}\bar{q}_{\substack{CD\\CC}} & 0 & 0 & 0 & \bar{p}_{\substack{CC\\CD}}q_{\substack{CD\\DC}} & 0 & 0 & 0 & \bar{p}_{\substack{CC\\CD}}\bar{q}_{\substack{CD\\CC}} & 0 & 0 & 0 \\
p_{\substack{CD\\CC}}q_{\substack{CC\\CC}} & 0 & 0 & 0 & p_{\substack{CD\\CC}}\bar{q}_{\substack{CC\\CC}} & 0 & 0 & 0 & \bar{p}_{\substack{CD\\CC}}q_{\substack{CC\\DC}} & 0 & 0 & 0 & \bar{p}_{\substack{CD\\CC}}\bar{q}_{\substack{CC\\DC}} & 0 & 0 & 0 \\
p_{\substack{CD\\CD}}q_{\substack{CD\\CC}} & 0 & 0 & 0 & p_{\substack{CD\\CD}}\bar{q}_{\substack{CD\\CC}} & 0 & 0 & 0 & \bar{p}_{\substack{CD\\CD}}q_{\substack{CD\\DC}} & 0 & 0 & 0 & \bar{p}_{\substack{CD\\CD}}\bar{q}_{\substack{CD\\DC}} & 0 & 0 & 0 \\
0 & p_{\substack{CC\\DC}}q_{\substack{DC\\CC}} & 0 & 0 & 0 & p_{\substack{CC\\DC}}\bar{q}_{\substack{DC\\CC}} & 0 & 0 & 0 & \bar{p}_{\substack{CC\\DC}}q_{\substack{DC\\DC}} & 0 & 0 & 0 & \bar{p}_{\substack{CC\\DC}}\bar{q}_{\substack{DC\\DC}} & 0 & 0 \\
0 & p_{\substack{CC\\DD}}q_{\substack{DD\\CC}} & 0 & 0 & 0 & p_{\substack{CC\\DD}}\bar{q}_{\substack{DD\\CC}} & 0 & 0 & 0 & \bar{p}_{\substack{CC\\DD}}q_{\substack{DD\\DC}} & 0 & 0 & 0 & \bar{p}_{\substack{CC\\DD}}\bar{q}_{\substack{DD\\DC}} & 0 & 0 \\
0 & p_{\substack{CD\\DC}}q_{\substack{DC\\CC}} & 0 & 0 & 0 & p_{\substack{CD\\DC}}\bar{q}_{\substack{DC\\CC}} & 0 & 0 & 0 & \bar{p}_{\substack{CD\\DC}}q_{\substack{DC\\DC}} & 0 & 0 & 0 & \bar{p}_{\substack{CD\\DC}}\bar{q}_{\substack{DC\\DC}} & 0 & 0 \\
0 & p_{\substack{CD\\DD}}q_{\substack{DD\\CC}} & 0 & 0 & 0 & p_{\substack{CD\\DD}}\bar{q}_{\substack{DD\\CC}} & 0 & 0 & 0 & \bar{p}_{\substack{CD\\DD}}q_{\substack{DD\\DC}} & 0 & 0 & 0 & \bar{p}_{\substack{CD\\DD}}\bar{q}_{\substack{DD\\DC}} & 0 & 0 \\
0 & 0 & p_{\substack{DC\\CC}}q_{\substack{CC\\CC}} & 0 & 0 & 0 & p_{\substack{DC\\CC}}\bar{q}_{\substack{CC\\CC}} & 0 & 0 & 0 & \bar{p}_{\substack{DC\\CC}}q_{\substack{CC\\DC}} & 0 & 0 & 0 & \bar{p}_{\substack{DC\\CC}}\bar{q}_{\substack{CC\\DC}} & 0 \\
0 & 0 & p_{\substack{DC\\CD}}q_{\substack{CD\\CD}} & 0 & 0 & 0 & p_{\substack{DC\\CD}}\bar{q}_{\substack{CD\\CD}} & 0 & 0 & 0 & \bar{p}_{\substack{DC\\CD}}q_{\substack{CD\\DD}} & 0 & 0 & 0 & \bar{p}_{\substack{DC\\CD}}\bar{q}_{\substack{CD\\DD}} & 0 \\
0 & 0 & p_{\substack{DD\\CC}}q_{\substack{CC\\CD}} & 0 & 0 & 0 & p_{\substack{DD\\CC}}\bar{q}_{\substack{CC\\CD}} & 0 & 0 & 0 & \bar{p}_{\substack{DD\\CC}}q_{\substack{CC\\DD}} & 0 & 0 & 0 & \bar{p}_{\substack{DD\\CC}}\bar{q}_{\substack{CC\\DD}} & 0 \\
0 & 0 & p_{\substack{DD\\CD}}q_{\substack{CD\\CD}} & 0 & 0 & 0 & p_{\substack{DD\\CD}}\bar{q}_{\substack{CD\\CD}} & 0 & 0 & 0 & \bar{p}_{\substack{DD\\CD}}q_{\substack{CD\\DD}} & 0 & 0 & 0 & \bar{p}_{\substack{DD\\CD}}\bar{q}_{\substack{CD\\DD}} & 0 \\
0 & 0 & 0 & p_{\substack{DC\\DC}}q_{\substack{DC\\CD}} & 0 & 0 & 0 & p_{\substack{DC\\DC}}\bar{q}_{\substack{DC\\CD}} & 0 & 0 & 0 & \bar{p}_{\substack{DC\\DC}}q_{\substack{DC\\DD}} & 0 & 0 & 0 & \bar{p}_{\substack{DC\\DC}}\bar{q}_{\substack{DC\\DD}} \\
0 & 0 & 0 & p_{\substack{DC\\DD}}q_{\substack{DD\\CD}} & 0 & 0 & 0 & p_{\substack{DC\\DD}}\bar{q}_{\substack{DD\\CD}} & 0 & 0 & 0 & \bar{p}_{\substack{DC\\DD}}q_{\substack{DD\\DD}} & 0 & 0 & 0 & \bar{p}_{\substack{DC\\DD}}\bar{q}_{\substack{DD\\DD}} \\
0 & 0 & 0 & p_{\substack{DD\\DC}}q_{\substack{DC\\CD}} & 0 & 0 & 0 & p_{\substack{DD\\DC}}\bar{q}_{\substack{DC\\CD}} & 0 & 0 & 0 & \bar{p}_{\substack{DD\\DC}}q_{\substack{DC\\DD}} & 0 & 0 & 0 & \bar{p}_{\substack{DD\\DC}}\bar{q}_{\substack{DC\\DD}} \\
0 & 0 & 0 & p_{\substack{DD\\DD}}q_{\substack{DD\\CD}} & 0 & 0 & 0 & p_{\substack{DD\\DD}}\bar{q}_{\substack{DD\\CD}} & 0 & 0 & 0 & \bar{p}_{\substack{DD\\DD}}q_{\substack{DD\\DD}} & 0 & 0 & 0 & \bar{p}_{\substack{DD\\DD}}\bar{q}_{\substack{DD\\DD}}
\end{pmatrix}. \tag{65}
$$

Given the transition matrix, we compute the invariant distribution of the respective Markov chain by solving $v = vM_A$. This invariant distribution is now a 16-dimensional vector,

$$
\mathbf{v} = \Big(v_{\substack{CC\\CC}}, v_{\substack{CC\\CD}}, v_{\substack{CD\\CC}}, v_{\substack{CD\\CD}}, v_{\substack{CC\\DC}}, v_{\substack{CC\\DD}}, v_{\substack{CD\\DC}}, v_{\substack{CD\\DD}}, v_{\substack{DC\\CC}}, v_{\substack{DC\\CD}}, v_{\substack{DD\\CC}}, v_{\substack{DD\\CD}}, v_{\substack{DC\\DC}}, v_{\substack{DC\\DD}}, v_{\substack{DD\\DC}}, v_{\substack{DD\\DD}}\Big). \tag{66}
$$

Using this invariant distribution, we calculate how often each player cooperates on average. To this end, we sum up over all outcomes in which the player cooperates in the last round,

$$
\begin{aligned}
\rho_1 &= v_{\substack{CC\\CC}} + v_{\substack{CC\\CD}} + v_{\substack{CD\\CC}} + v_{\substack{CD\\CD}} + v_{\substack{CC\\DC}} + v_{\substack{CC\\DD}} + v_{\substack{CD\\DC}} + v_{\substack{CD\\DD}}, \\
\rho_2 &= v_{\substack{CC\\CC}} + v_{\substack{CC\\CD}} + v_{\substack{CD\\CC}} + v_{\substack{CD\\CD}} + v_{\substack{DC\\CC}} + v_{\substack{DC\\CD}} + v_{\substack{DD\\CC}} + v_{\substack{DD\\CD}}.
\end{aligned} \tag{67}
$$

Given these average cooperation rates, the players' payoffs are

$$
\pi_1 = b\rho_2 - c\rho_1 \quad \text{and} \quad \pi_2 = b\rho_1 - c\rho_2. \tag{68}
$$

Stability of pure memory-2 strategies. In Hilbe *et al*[1], the authors introduce an algorithm to describe the Nash equilibria of the simultaneous game among all pure memory-2 strategies. In addition, this algorithm outputs the range for the benefit-to-cost ratio b/c for which the respective strategy is an equilibrium. In

the following, we briefly recapitulate that algorithm and apply it to the alternating game.

To test whether a given pure strategy $\mathbf{p}$ is stable, we first compute the average probability ρ with which the strategy cooperates against itself, using Eq. (67). In particular, the payoff of two players who both use strategy $\mathbf{p}$ is $\pi = b\rho - c\rho$. Now, if one player instead switches to some other pure strategy $\mathbf{q}$, the payoff of the deviating player is $\tilde{\pi}_{\mathbf{q}} = b\tilde{\rho}_{\mathbf{p}} - c\tilde{\rho}_{\mathbf{q}}$. Here, $\tilde{\rho}_{\mathbf{p}}$ is the average cooperation probability of the $\mathbf{p}$-player, and $\tilde{\rho}_{\mathbf{q}}$ is the cooperation probability of the $\mathbf{q}$-player. For $(\mathbf{p}, \mathbf{p})$ to be a Nash equilibrium, it needs to be the case that $\pi \geq \tilde{\pi}_{\mathbf{q}}$. This condition simplifies to

$$b \cdot x_{\mathbf{p},\mathbf{q}} \geq c \cdot y_{\mathbf{q},\mathbf{p}}, \tag{69}$$

with $x_{\mathbf{p},\mathbf{q}} := \rho - \tilde{\rho}_{\mathbf{p}}$ and $y_{\mathbf{q},\mathbf{p}} := \rho - \tilde{\rho}_{\mathbf{q}}$. Depending on $x_{\mathbf{p},\mathbf{q}}$ and $y_{\mathbf{q},\mathbf{p}}$, there are four possible cases.

1. If $x_{\mathbf{p},\mathbf{q}} > 0$ and $y_{\mathbf{q},\mathbf{p}} > 0$, condition (69) is satisfied if and only if $b/c \geq y_{\mathbf{q},\mathbf{p}}/x_{\mathbf{p},\mathbf{q}}$.

2. If $x_{\mathbf{p},\mathbf{q}} < 0$ and $y_{\mathbf{q},\mathbf{p}} \leq 0$, condition (69) is satisfied if and only if $b/c \leq y_{\mathbf{q},\mathbf{p}}/x_{\mathbf{p},\mathbf{q}}$.

3. If $x_{\mathbf{p},\mathbf{q}} \leq 0$ and $y_{\mathbf{q},\mathbf{p}} > 0$, condition (69) is never satisfied.

4. If $x_{\mathbf{p},\mathbf{q}} \geq 0$ and $y_{\mathbf{q},\mathbf{p}} \leq 0$, condition (69) is always satisfied.

Based on these considerations, we define the following three subsets of memory-2 strategies with respect to the focal strategy $\mathbf{p}$,

$$\begin{aligned} Q_1(\mathbf{p}) &= \big\{\mathbf{q} \;\big|\; x_{\mathbf{p},\mathbf{q}} > 0 \text{ and } y_{\mathbf{q},\mathbf{p}} > 0\big\}, \\ Q_2(\mathbf{p}) &= \big\{\mathbf{q} \;\big|\; x_{\mathbf{p},\mathbf{q}} < 0 \text{ and } y_{\mathbf{q},\mathbf{p}} \leq 0\big\}, \\ Q_3(\mathbf{p}) &= \big\{\mathbf{q} \;\big|\; x_{\mathbf{p},\mathbf{q}} \leq 0 \text{ and } y_{\mathbf{q},\mathbf{p}} > 0\big\}. \end{aligned} \tag{70}$$

Taking into account the four cases described above, the first set $Q_1(\mathbf{p})$ contains all memory-2 strategies against which $\mathbf{p}$ is only stable if b/c is sufficiently large. The second set $Q_2(\mathbf{p})$ contains all memory-2 strategies against which $\mathbf{p}$ is only stable if b/c is sufficiently small. The last set contains the strategies against which $\mathbf{p}$ is never stable, for no b/c. In particular, we can use these sets to define lower und upper bounds for the benefit-to-cost ratio for $\mathbf{p}$ to be a Nash equilibrium,

$$(b/c)_{LB} = \max\big\{\, y_{\mathbf{q},\mathbf{p}}/x_{\mathbf{p},\mathbf{q}} \;\big|\; \mathbf{q} \in Q_1(\mathbf{p})\big\} \quad \text{and} \quad (b/c)_{UB} = \min\big\{\, y_{\mathbf{q},\mathbf{p}}/x_{\mathbf{p},\mathbf{q}} \;\big|\; \mathbf{q} \in Q_2(\mathbf{p})\big\}. \tag{71}$$

Using these thresholds, it follows that $\mathbf{p}$ can only be a Nash equilibrium if

$$(b/c)_{LB} \leq b/c \leq (b/c)_{UB} \quad \text{and} \quad Q_3(\mathbf{p}) = \emptyset. \tag{72}$$

For a given strategy $\mathbf{p}$, these conditions can be checked by computing $x_{\mathbf{p},\mathbf{q}}$ and $y_{\mathbf{q},\mathbf{p}}$ for all 2^{16} pure memory-2 strategies $\mathbf{q}$. In **Supplementary Fig. 7a**, we illustrate the result of this algorithm for both the simultaneous game and the alternating game. For that figure, we call a Nash equilibrium locally robust if it is an equilibrium for a substantial portion of the parameter space; specifically, we require

$(b/c)_{UB} - (b/c)_{LB} > 0.2$. The figure then displays all locally robust Nash equilibria for $b/c \leq 5$. We find that in the simultaneous game, there are 34 such equilibria. Out of those, there are several equilibria that yield very little cooperation (colored in red). These equilibrium strategies include, for example, *ALLD*. On the other hand, for $b/c > 3/2$, there are also several equilibria that yield almost full cooperation. These strategies display so-called all-or-none behavior[1,30]. Players with these AON_k strategies tend to cooperate if in each of the past k rounds, either both players cooperated or no one did.

For the alternating game, we find that the only locally robust Nash equilibrium among the pure memory-2 strategies is *ALLD*. There are two additional strategies that are Nash equilibria without being locally robust. These are:

$$\begin{aligned} \mathbf{p}_1 &= (0,\ 0,\ 0,\ 0,\ 1\ ,0,\ 1\ ,0,\ 1,\ 0,\ 0,\ 1,\ 0,\ 1,\ 0,\ 1) \\ \mathbf{p}_2 &= (1,\ 1,\ 1,\ 1,\ 0,\ 0,\ 0,\ 0,\ 1,\ 1,\ 0,\ 1,\ 0,\ 0,\ 0,\ 0) \end{aligned} \tag{73}$$

Both of these strategies are only stable for a single value of b/c. For the given error rate of $\varepsilon = 0.02$, this value is $b/c = 4.1464$ for the first strategy and $b/c = 1.04166$ for the second. Moreover, none of the two strategies is self-cooperative. The cooperation rate of the first strategy against itself is 64.9%; the self-cooperation rate of the second strategy is 50.0%.

These results show that there is no Nash equilibrium among the pure memory-2 strategies that can sustain full cooperation in the alternating game. Since any evolutionarily stable strategy needs to be a pure strategy, we conclude that evolutionarily stable cooperation in the alternating game is infeasible (as in the case of the memory-1 strategies).

Evolutionary dynamics. In addition to these static results, we have also explored the evolutionary dynamics among pure memory-2 strategies with simulations. For these simulations we consider the case of an infinitely repeated game with a positive error rate ($\varepsilon = 0.02$) in the limit of rare mutations. **Supplementary Fig. 7b** shows the evolving average cooperation rate for both the simultaneous and the alternating game (averaged over 100 independent simulation runs for $b = 3$). Although only the simultaneous game has fully cooperative Nash equilibria, the two scenarios lead to largely comparable overall cooperation rates. We obtain similar results for other benefit values (**Supplementary Fig. 7c**).

To explore this result in more detail, **Supplementary Fig. 7d** analyzes the players' average cooperation probabilities when players adopt a self-cooperative strategy. In the simultaneous game, the player's average cooperation probabilities resemble the typical behavior of AON_2 strategies: players have a high cooperation probability if either (*i*) both players mutually cooperated for two rounds, (*ii*) if both players mutually defected for two rounds, or (*iii*) if both players cooperated in the last round but defected in the second-to-last round. In all other cases, the conditional cooperation probability is below 50%. In contrast, in the alternating game, players seem to be prepared to cooperate as long as the co-player did not defect more often than the focal player did.

However, **Supplementary Fig. 7e** shows that in line with the equilibrium analysis, the self-cooperative strategies in the simultaneous game tend to be more robust. For this panel, we have recorded for each

self-cooperative strategy adopted by the resident population how many mutant strategies it takes on average until the first mutant reaches fixation. We find that in the simultaneous game, it takes on average almost 3,000 mutant strategies to invade a self-cooperative resident. In the alternating game, this number is considerably lower; on average it takes less than 800 random mutant strategies until the resident strategy is successfully invaded. Overall, these results suggest that cooperation in the simultaneous game is generally more robust. However, also in the alternating game, individuals adopt self-cooperative strategies for a substantial amount of time.

Games in spatial populations

Motivation. The simulation results for the baseline model and the previous model extensions are based on the assumption that the population is well-mixed. This assumption has two consequences. First, when playing games, all members of the population are equally likely to interact with everyone else. Second, for the evolutionary updating, all population members are equally likely to act as a role model for any given focal player. The assumption of well-mixed populations has a long tradition in evolutionary game theory (see, for instance, the text books in Refs. 8, 31). However, there is also a rich literature asking how strategies spread in structured populations[32–35]. In the following, we thus explore the strategies that evolve when both interactions and imitation events are local.

To explore the dynamics of spatial games, we closely follow the approach of Brauchli *et al*[36]. They study the simultaneous game on a square lattice with periodic boundary conditions. The set of available strategies consists of all (stochastic) memory-1 strategies. With extensive computer simulations, the study shows that spatial games are generally more conducive to the evolution of cooperation. Moreover, evolutionary trajectories are less chaotic, and more likely to result in eventual behavior that is consistent with the strategy Win-Stay Lose-Shift. In the following, we use their setup to (*i*) repeat their simulations for the simultaneous game, and (*ii*) extend these simulations to the case of alternating games.

Model setup. For our exploration of games in structured populations, we consider a population with 2,500 individuals placed on a 50×50 square lattice. Individuals use memory-1 strategies to engage in a repeated game with each of their eight immediate neighbors (we use a Moore neighborhood with periodic boundary conditions). We consider two independent scenarios. These scenarios differ in whether the game being played is the simultaneous game or the alternating game. In both cases we use the baseline versions of these games (in which there is no discounting of the future). A player's payoff at any point in time is defined as the player's average payoff against its eight neighbors (taking the sum of the eight pairwise payoffs would yield the same result).

Initially, we assume that all population members adopt the strategy *ALLD*. In each generation of the simulation, all population members update their strategies. With probability $1-\mu$, an individual who is to update its strategy adopts the strategy of the neighbor with the highest payoff. With the converse probability μ, the individual adopts a random memory-1 strategy, by drawing four numbers

$(p_{CC}, p_{CD}, p_{DC}, p_{DD})$ from the hypercube $[0,1]^4$ uniformly at random. This elementary process is then repeated for $20,000$ generations. For each generation, we record the strategy that is adopted by each individual, and the average cooperation rate across all interactions taking place in the population.

This overall setup agrees with the setup considered by Brauchli *et al*[36], with a few minor exceptions. First, we use a different initial population, *ALLD*, to better visualize the emergence of cooperation in a population of defectors. In contrast, Brauchli *et al*[36] assume that all players initially use the perfectly random strategy $\mathbf{p} = (0.5, 0.5, 0.5, 0.5)$. Second, because we are also interested in the outcome of alternating game, we use one-shot payoffs based on the infinitely repeated donation game, as defined by Eq. (7). Brauchli *et al*[36] instead use the payoff values of Axelrod[37], and they consider games that last on average between 100 and 200 rounds. Despite these differences, our simulation results for the simultaneous game are comparable to theirs (as described in more detail below).

Evolutionary dynamics. For our evolutionary simulations, we used parameters that are comparably hostile to cooperation: the benefit of cooperation is smaller than in previous simulations (now $b=2$ instead of $b=3$), and errors occur at an appreciable rate, $\varepsilon = 0.02$. **Fig. 6g** shows the resulting cooperation dynamics (averaged over 50 independent simulations). Despite the hostile conditions, we observe that spatial games lead to predominantly cooperative populations rather quickly. When we compare the simultaneous game to the alternating game, we observe that the simultaneous game leads to more (and to more robust) cooperation. To explore these results in more detail, **Fig. 6h** shows snapshots of the population at different points in time. In the simultaneous game, we observe that populations almost always converge to a largely homogeneous configuration of cooperative players. In contrast, in the alternating game, different simulation runs can exhibit very different behaviors. In some simulations, we observe a similar dynamics towards almost uniform cooperation as in the simultaneous game. Other simulation runs, however, result in stable mixtures of cooperating and defecting players (this latter case is displayed in the bottom panel of **Fig. 6h**).

In a next step, we explored which strategies the players use to maintain cooperation in the two scenarios. To this end, we recorded all used strategies that yield a cooperation rate of at least 80% against themselves; then we computed the respective average cooperation probabilities across all these strategies (**Fig. 6i**). For the simultaneous game, this average strategy exhibits the characteristics of Win-Stay Lose-Shift (as already reported by Brauchli *et al*[36]). Players are most likely to cooperate after mutual cooperation and mutual defection; after all other outcomes, they tend to defect. For the alternating game, the average strategy reflects some of the characteristics of Stochastic Firm-but-Fair. Here again, players are most likely to cooperate if the opponent's last move was to cooperate.

Overall, our results are in line with the main conclusions of the baseline model: Cooperation in alternating games is slightly less robust, and it requires different kinds of strategies. At the same time, the simulations also highlight the intriguing spatial patterns that can arise in structured populations.

The effect of local mutations

Motivation. For our simulations so far, we assumed that when a mutation occurs, the player's new strategy can be arbitrarily different from the player's present strategy. In that case we speak of 'global mutations'. The assumption of global mutations is fairly common in the evolutionary game theory literature[12,28,38,39]. However, there is also important work on the effects of local mutations[40,41]. When mutations are local, they only lead to a slight modification of the players' strategies. Which of the two mutation schemes is more relevant depends on the type of evolution considered. Biological evolution is perhaps better described by local mutations, whereas for cultural processes global mutations may be more reasonable.

Compared to global mutations, local mutations can affect the dynamics in three ways:

1. It can introduce additional (local) equilibria. The corresponding strategies are robust with respect to local mutants, although they can be invaded by strategies further away in the strategy space;
2. It affects how likely any given equilibrium is reached;
3. It affects the robustness of any given equilibrium: When mutations have a sufficiently short range, *any* mutant strategy has approximately the same payoff as the resident strategy (since payoffs are continuous in the players' strategies). As a result, even a strategy that is evolutionarily stable can be invaded by a strategy that is sufficiently close-by with an approximate probability of $1/N$ (the neutral fixation probability)[42]. As a result, the concept of evolutionary stability becomes overall less relevant to describe the stochastic evolutionary dynamics in finite populations with local mutations.

To explore these effects in the context of alternating games, we have implemented additional simulations.

Model setup. We use the same basic framework that we used to describe the evolutionary dynamics of the baseline model. However, this time, when a player with strategy $\mathbf{p}$ undergoes a mutation, the new strategy is uniformly chosen among all memory-1 strategies $\mathbf{p}'$ that satisfy

$$\left|p'_{ij} - p_{ij}\right| \leq m, \quad \text{for all } i,j \in \{C,D\}. \tag{74}$$

We refer to the parameter m as the mutation range; it describes how far apart the mutant strategy can be from the parent strategy. For $m \geq 1$, we recover the case of global mutations. For smaller m, mutations are restricted to generate strategies in a local neighborhood of the parent strategy.

Evolutionary dynamics. In **Supplementary Fig. 8a,b**, we compare the results for global mutations with the corresponding results for local mutations (using $m = 0.05$). We observe that for both the simultaneous and the alternating game, overall cooperation rates under local mutations tend to be lower on average.

However, the magnitude of the effect differs: While local mutations strongly reduce cooperation in the simultaneous game, it has a much smaller negative effect on the alternating game.

To analyze this effect in more detail, we again compute an average over all self-cooperative strategies used by the players (**Supplementary Fig. 8c,d**). The resulting average strategies resemble Win-Stay Lose-Shift (in the simultaneous game) and Stochastic Firm-but-Fair (in the alternating game), largely independent of whether mutations are local or global. However, local mutations have a substantial effect on the robustness of these self-cooperative strategies. For local mutations, the number of mutants it takes to invade a resident self-cooperative strategy is of the order of N (which is 100 in these simulations), as expected. In contrast, under global mutations, resident strategies typically resist $\sim 6,300$ mutant strategies in the simultaneous game, and $\sim 1,600$ mutant strategies in the alternating game.

In **Supplementary Fig. 8e,f**, we systematically explore the effect of different mutation ranges between $m = 0.05$ (local mutations) and $m = 0.95$ (almost global mutations). The alternating game yields slightly more cooperation than the simultaneous game when mutations are local. However, once the mutation range exceeds $m \approx 0.4$, it is the simultaneous game that is more conducive to cooperation.

Supplementary Note 4: Proofs of the analytical results

Proof of Proposition 1. Let $\mathbf{v}(\mathbf{p},\mathbf{q})$ be an invariant distribution of the game between strategies $\mathbf{p}$ and $\mathbf{q}$. Then $\mathbf{v}(\mathbf{p},\mathbf{q})$ is a solution of the equation $\mathbf{v} = \mathbf{v} M_A(\mathbf{p},\mathbf{q})$, with $M_A(\mathbf{p},\mathbf{q})$ being the transition matrix of the game as defined by Eq. (5). More explicitly, $\mathbf{v}(\mathbf{p},\mathbf{q})$ solves the following system of linear equations,

$$\begin{aligned}
v_{CC} &= v_{CC}\, p_{CC}\, q_{CC} + v_{CD}\, p_{CD}\, q_{DC} + v_{DC}\, p_{DC}\, q_{CC} + v_{DD}\, p_{DD}\, q_{DC}\\
v_{CD} &= v_{CC}\, p_{CC}\,(1-q_{CC}) + v_{CD}\, p_{CD}\,(1-q_{DC}) + v_{DC}\, p_{DC}\,(1-q_{CC}) + v_{DD}\, p_{DD}\,(1-q_{DD})\\
v_{DC} &= v_{CC}\,(1-p_{CC})\, q_{CD} + v_{CD}\,(1-p_{CD})\, q_{DD} + v_{DC}\,(1-p_{DC})\, q_{CD} + v_{DD}\,(1-p_{DD})\, q_{DD}\\
v_{DD} &= v_{CC}\,(1-p_{CC})\,(1-q_{CD}) + v_{CD}\,(1-p_{CD})\,(1-q_{DD}) + v_{DC}\,(1-p_{DC})\,(1-q_{CD}) + v_{DD}\,(1-p_{DD})\,(1-q_{DD}).
\end{aligned} \tag{75}$$

By simplifying the right hand's side, we can write Eq. (75) as

$$\begin{aligned}
v_{CC} &= (v_{CC}\, p_{CC} + v_{DC}\, p_{DC})\, q_{CC} + (v_{CD}\, p_{CD} + v_{DD}\, p_{DD})\, q_{DC}\\
v_{CD} &= (v_{CC}\, p_{CC} + v_{DC}\, p_{DC})\,(1-q_{CC}) + (v_{CD}\, p_{CD} + v_{DD}\, p_{DD})\,(1-q_{DC})\\
v_{DC} &= \big(v_{CC}\,(1-p_{CC}) + v_{DC}\,(1-p_{DC})\big)\, q_{CD} + \big(v_{CD}\,(1-p_{CD}) + v_{DD}\,(1-p_{DD})\big)\, q_{DD}\\
v_{DD} &= \big(v_{CC}\,(1-p_{CC}) + v_{DC}\,(1-p_{DC})\big)\,(1-q_{CD}) + \big(v_{CD}\,(1-p_{CD}) + v_{DD}\,(1-p_{DD})\big)\,(1-q_{DD}).
\end{aligned} \tag{76}$$

Now by using assumption (9)

$$\begin{aligned}
(v_{CC}+v_{DC})\,\tilde{p}_C &= v_{CC}\, p_{CC} + v_{DC}\, p_{DC}\\
(v_{CD}+v_{DD})\,\tilde{p}_D &= v_{CD}\, p_{CD} + v_{DD}\, p_{DD},
\end{aligned} \tag{77}$$

and its equivalent formulation

$$\begin{aligned}(v_{CC}+v_{DC})\,(1-\tilde{p}_C) &= v_{CC}\,(1-p_{CC}) + v_{DC}\,(1-p_{DC})\\ (v_{CD}+v_{DD})\,(1-\tilde{p}_D) &= v_{CD}\,(1-p_{CD}) + v_{DD}\,(1-p_{DD}),\end{aligned} \tag{78}$$

we can write Eq. (76) as

$$\begin{aligned}v_{CC} &= (v_{CC}+v_{DC})\,\tilde{p}_C\,q_{CC} &&+ (v_{CD}+v_{DD})\,\tilde{p}_D\,q_{DC}\\ v_{CD} &= (v_{CC}+v_{DC})\,\tilde{p}_C\,(1-q_{CC}) &&+ (v_{CD}+v_{DD})\,\tilde{p}_D\,(1-q_{DC})\\ v_{DC} &= (v_{CC}+v_{DC})\,(1-\tilde{p}_C)\,q_{CD} &&+ (v_{CD}+v_{DD})\,(1-\tilde{p}_D)\,q_{DD}\\ v_{DD} &= (v_{CC}+v_{DC})\,(1-\tilde{p}_C)\,(1-q_{CD}) &&+ (v_{CD}+v_{DD})\,(1-\tilde{p}_D)\,(1-q_{DD}).\end{aligned} \tag{79}$$

This equation can be rewritten as $\mathbf{v} = \mathbf{v}M_A(\tilde{\mathbf{p}}, \mathbf{q})$, where $M_A(\tilde{\mathbf{p}}, \mathbf{q})$ is now the transition matrix of the game between $\tilde{\mathbf{p}} = (\tilde{p}_C, \tilde{p}_D)$ and $\mathbf{q}$. If $\mathbf{v}$ solves $\mathbf{v} = \mathbf{v}\,M_A(\mathbf{p}, \mathbf{q})$, it thus also solves $\mathbf{v} = \mathbf{v}M_A(\tilde{\mathbf{p}}, \mathbf{q})$. □

Proof of Proposition 2. Consider an alternating game in which both players' strategies are fixed and player 2 adopts a memory-1 strategy $\mathbf{q}$. Let $v_{a_1,a_2}(t)$ denote the probability that the players choose the actions $(a_1, a_2) \in \{CC, CD, DC, DD\}$ at time t in the resulting game. By assumption, the following limiting averages are well-defined,

$$v_{CC} = \lim_{T\to\infty} \frac{1}{T}\sum_{t=1}^{T} v_{CC}(t), \;\; v_{CD} = \lim_{T\to\infty} \frac{1}{T}\sum_{t=1}^{T} v_{CD}(t), \;\; v_{DC} = \lim_{T\to\infty} \frac{1}{T}\sum_{t=1}^{T} v_{DC}(t), \;\; v_{DD} = \lim_{T\to\infty} \frac{1}{T}\sum_{t=1}^{T} v_{DD}(t). \tag{80}$$

We write these four limits as a vector $\mathbf{v} = (v_{CC}, v_{CD}, v_{DC}, v_{DD})$. Moreover, let $p_{a_1,a_2}(t)$ denote the conditional probability that player 1 cooperates at time $t+1$, given the history of the game is such that the players' actions at time t are (a_1, a_2). Again, by assumption we can define a reactive strategy $\tilde{\mathbf{p}} = (\tilde{p}_C, \tilde{p}_D)$ as an implicit solution of two equations

$$\begin{aligned}(v_{CC}+v_{DC})\,\tilde{p}_C &= \lim_{T\to\infty} \frac{1}{T}\sum_{t=1}^{T} v_{CC}(t)\,p_{CC}(t) + v_{DC}(t)\,p_{DC}(t)\\ (v_{CD}+v_{DD})\,\tilde{p}_D &= \lim_{T\to\infty} \frac{1}{T}\sum_{t=1}^{T} v_{CD}(t)\,p_{CD}(t) + v_{DD}(t)\,p_{DD}(t).\end{aligned} \tag{81}$$

We need to show that the vector $\mathbf{v}$ satisfies the linear system $\mathbf{v} = \mathbf{v}M_A(\tilde{\mathbf{p}}, \mathbf{q})$, where $M_A(\tilde{\mathbf{p}}, \mathbf{q})$ is the transition matrix defined by Eq. (5). We show this for the first equation of the system; all other equations are verified analogously. By the definition of $v_{a_1,a_2}(t)$ and $p_{a_1,a_2}(t)$, we can write $v_{CC}(t+1)$ as follows,

$$v_{CC}(t+1) = v_{CC}(t)\,p_{CC}(t)\,q_{CC} + v_{CD}(t)\,p_{CD}(t)\,q_{DC} + v_{DC}(t)\,p_{DC}(t)\,q_{CC} + v_{DD}(t)\,p_{DD}(t)\,q_{DC} \tag{82}$$

By summing up this equation for the first T time steps and collecting terms on the right hand side, we obtain

$$\sum_{t=1}^{T} v_{CC}(t+1) = \left(\sum_{t=1}^{T} v_{CC}(t)\, p_{CC}(t) + v_{DC}(t)\, p_{DC}(t)\right) q_{CC} + \left(\sum_{t=1}^{T} v_{CD}(t)\, p_{CD}(t) + v_{DD}(t)\, p_{DD}(t)\right) q_{DC} \tag{83}$$

Dividing both sides by T, taking the limit $T \to \infty$, and replacing the limits by the respective expressions in Eqs. (80) and (81), we obtain

$$v_{CC} = (v_{CC}+v_{DC})\,\tilde{p}_C\, q_{CC} + (v_{CD}+v_{DD})\,\tilde{p}_D\, q_{DC}. \tag{84}$$

This is exactly the first equation of the linear system $\mathbf{v} = \mathbf{v}M_A(\tilde{\mathbf{p}}, \mathbf{q})$. □

Proof of Lemma 1. The payoff equation (14) follows immediately from the formula in Eq. (7) when using the strategies $\mathbf{p}$ and $\mathbf{q}$ as input. To show the monotonicity property in p_C, we keep $\mathbf{q}$ and p_D fixed and consider the function $p_C \mapsto f_C(p_C) := \pi(\mathbf{p}, \mathbf{q})$. By Eq. (14), this function can be written as

$$f_C(p_C) = \frac{a_1 + a_2\, p_C}{a_3 + a_4\, p_C}, \tag{85}$$

where a_1, a_2, a_3, a_4 are constants that are independent of p_C. Calculating the derivative yields

$$\frac{\partial f_C}{\partial p_C} = \frac{a_2 a_3 - a_1 a_4}{(a_3 + a_4\, p_C)^2}. \tag{86}$$

In particular, the sign of the derivative is independent of p_C. That is, $f_C(p_C) = \pi(\mathbf{p}, \mathbf{q})$ is either strictly increasing (if $a_2 a_3 > a_1 a_4$), strictly decreasing (if $a_2 a_3 < a_1 a_4$), or constant in p_C (if $a_2 a_3 = a_1 a_4$). A similar argument shows that also the map $p_D \mapsto f_D(p_D) := \pi(\mathbf{p}, \mathbf{q})$ is monotonic in p_D. □

Proof of Proposition 3. The proof is by iterated application of **Lemma 1**. Let $\mathbf{q}$ and $\mathbf{p}$ be arbitrary but fixed. We iteratively define $\mathbf{p^0} = (p_C^0, p_D^0) := (p_C, p_D)$,

$$\mathbf{p^1} = \begin{cases} (1, p_D^0) & \text{if } \pi\big((1, p_D^0), \mathbf{q}\big) \geq \pi(\mathbf{p}, \mathbf{q}) \\ (0, p_D^0) & \text{otherwise,} \end{cases} \tag{87}$$

and

$$\mathbf{p^2} = \begin{cases} (p_C^1, 1) & \text{if } \pi\big((p_C^1, 1), \mathbf{q}\big) \geq \pi(\mathbf{p}, \mathbf{q}) \\ (p_C^1, 0) & \text{otherwise} \end{cases} \tag{88}$$

Because we only change one component in each step, it follows by the monotonicity property in **Lemma 1** that $\pi(\mathbf{p^2},\mathbf{q}) \geq \pi(\mathbf{p^1},\mathbf{q}) \geq \pi(\mathbf{p^0},\mathbf{q})$. Moreover, $\mathbf{p^2} \in \{0,1\}^2$ by construction. Therefore, defining $\mathbf{p}' := \mathbf{p^2}$ yields the desired result. □

Proof of Lemma 2.

1. Suppose $\mathbf{q}$ is a generic Nash equilibrium and $\tilde{\mathbf{q}} = (\tilde{q}_C, \tilde{q}_D) \in (0,1)^2$ is its reactive marginalization with respect to itself. In particular, $\tilde{\mathbf{q}}$ is a generic best response to $\mathbf{q}$, since $\mathbf{q}$ is a best response to itself. It follows that the map $\tilde{q}_C \mapsto \pi(\tilde{\mathbf{q}},\mathbf{q})$ needs to be constant (otherwise **Lemma 1** implies that it is either strictly increasing or decreasing, which both contradicts the best reply property). As a consequence, both respective boundary strategies $\tilde{\mathbf{q}}' := (0,\tilde{q}_D)$ and $\tilde{\mathbf{q}}'' := (1,\tilde{q}_D)$ are also generic best responses to $\mathbf{q}$. With the same argument, one can now show that the maps $\tilde{q}_D \mapsto \pi(\tilde{\mathbf{q}}',\mathbf{q})$ and $\tilde{q}_D \mapsto \pi(\tilde{\mathbf{q}}'',\mathbf{q})$ are also constant. Therefore all respective boundary strategies – *ALLD*, *ATFT*, *TFT*, *ALLC* – are generic best responses to $\mathbf{q}$. In particular, all four boundary strategies yield the same payoff against $\mathbf{q}$. That is, we have shown Eq. (20). Because Eq. (20) implies Eq. (21), we conclude that $\mathbf{q}$ is an equalizer.

2. Using Eqs. (6) and (10), we can compute the reactive marginalization $\tilde{\mathbf{q}}$ of $\mathbf{q}$ with respect to itself explicitly. This yields

$$\tilde{\mathbf{q}} = (\tilde{q}_C, \tilde{q}_D) = \left(\frac{q_{DC}}{1-q_{CC}+q_{DC}}, \frac{q_{DD}}{1-q_{CD}+q_{DD}}\right) \tag{89}$$

By assumption, this reactive marginalization is either semi-stochastic or deterministic, and therefore either $\tilde{q}_C \in \{0,1\}$, $\tilde{q}_D \in \{0,1\}$, or both. This gives rise to four possible cases,

$$\begin{aligned} \tilde{q}_C \in \{0,1\} \quad &\Leftrightarrow \quad \mathbf{q} = (1, q_{CD}, q_{DC}, q_{DD}) \quad \text{or} \quad \mathbf{q} = (q_{CC}, q_{CD}, 0, q_{DD}) \\ \tilde{q}_D \in \{0,1\} \quad &\Leftrightarrow \quad \mathbf{q} = (q_{CC}, 1, q_{DC}, q_{DD}) \quad \text{or} \quad \mathbf{q} = (q_{CC}, q_{CD}, q_{DC}, 0). \end{aligned} \tag{90}$$

The first and the last case, $\mathbf{q} = (1, q_{CD}, q_{DC}, q_{DD})$ and $\mathbf{q} = (q_{CC}, q_{CD}, q_{DC}, 0)$ correspond to the self-cooperating and self-defecting players, respectively. They give rise to a generic Nash equilibrium if and only if the respective conditions for being a partner, or for being a defector are satisfied, as given by Eqs. (18) and (19). In the following, we discuss the remaining two cases. For those we can assume without loss of generality that $q_{CC} < 1$ and $q_{DD} > 0$.

First, suppose that $\mathbf{q} = (q_{CC}, q_{CD}, 0, q_{DD})$. Then by Eq. (15), the payoff of $\mathbf{q}$ against itself is

$$\pi(\mathbf{q},\mathbf{q}) = \frac{q_{DD}}{1-q_{CD}+2q_{DD}}\,(b-c). \tag{91}$$

If a player deviates to *ALLD* instead, its payoff according to Eq. (16) becomes

$$\pi(ALLD, \mathbf{q}) = \frac{q_{DD}}{1 - q_{CD} + q_{DD}} \cdot b. \tag{92}$$

In particular, $\pi(ALLD, \mathbf{q}) > \pi(\mathbf{q}, \mathbf{q})$.

Second, suppose $\mathbf{q} = (q_{CC}, 1, q_{DC}, q_{DD})$. Again we use Eq. (15) to compute the payoff of $\mathbf{q}$ against itself, which yields

$$\pi(\mathbf{q}, \mathbf{q}) = \frac{1 - q_{CC} + q_{DC}}{2(1 - q_{CC}) + q_{DC}} \,(b-c). \tag{93}$$

However, if a player deviates to *ALLD*, its payoff becomes $\pi(ALLD, \mathbf{q}) = b > \pi(\mathbf{q}, \mathbf{q})$.

□

Proof of Proposition 4.

Both results follow from a straightforward application of our earlier results for the case without errors. For (1), we note that

$$\begin{aligned}
\mathbf{v}^\varepsilon(\tilde{\mathbf{p}}, \mathbf{q}) &\overset{\text{Eq. (26)}}{=} \mathbf{v}(\tilde{\mathbf{p}}^\varepsilon, \mathbf{q}^\varepsilon) \\
&\overset{\text{Eq. (23)}}{=} \mathbf{v}\Big(\big(\tfrac{v^\varepsilon_{CC}(\mathbf{p},\mathbf{q})\,p^\varepsilon_{CC} + v^\varepsilon_{DC}(\mathbf{p},\mathbf{q})\,p^\varepsilon_{DC}}{v^\varepsilon_{CC}(\mathbf{p},\mathbf{q}) + v^\varepsilon_{DC}(\mathbf{p},\mathbf{q})}, \tfrac{v^\varepsilon_{CD}(\mathbf{p},\mathbf{q})\,p^\varepsilon_{CD} + v^\varepsilon_{DD}(\mathbf{p},\mathbf{q})\,p^\varepsilon_{DD}}{v^\varepsilon_{CD}(\mathbf{p},\mathbf{q}) + v^\varepsilon_{DD}(\mathbf{p},\mathbf{q})}\big)\,,\ \mathbf{q}^\varepsilon\Big) \\
&\overset{\text{Eq. (26)}}{=} \mathbf{v}\Big(\big(\tfrac{v_{CC}(\mathbf{p}^\varepsilon,\mathbf{q}^\varepsilon)\,p^\varepsilon_{CC} + v_{DC}(\mathbf{p}^\varepsilon,\mathbf{q}^\varepsilon)\,p^\varepsilon_{DC}}{v_{CC}(\mathbf{p}^\varepsilon,\mathbf{q}^\varepsilon) + v_{DC}(\mathbf{p}^\varepsilon,\mathbf{q}^\varepsilon)}, \tfrac{v_{CD}(\mathbf{p}^\varepsilon,\mathbf{q}^\varepsilon)\,p^\varepsilon_{CD} + v_{DD}(\mathbf{p}^\varepsilon,\mathbf{q}^\varepsilon)\,p^\varepsilon_{DD}}{v_{CD}(\mathbf{p}^\varepsilon,\mathbf{q}^\varepsilon) + v_{DD}(\mathbf{p}^\varepsilon,\mathbf{q}^\varepsilon)}\big)\,,\ \mathbf{q}^\varepsilon\Big) \\
&\overset{\text{Prop. 1}}{=} \mathbf{v}\big(\mathbf{p}^\varepsilon, \mathbf{q}^\varepsilon\big) \\
&\overset{\text{Eq. (26)}}{=} \mathbf{v}^\varepsilon(\mathbf{p}, \mathbf{q}).
\end{aligned} \tag{94}$$

For (2), we note that by their definition in Eq. (29), $\tilde{p}^\varepsilon_C$ and $\tilde{p}^\varepsilon_D$ satisfy the conditions (11) in **Proposition 2**. Therefore we can conclude

$$\mathbf{v}^\varepsilon(\tilde{\mathbf{p}}, \mathbf{q}) \overset{\text{Eq. (26)}}{=} \mathbf{v}(\tilde{\mathbf{p}}^\varepsilon, \mathbf{q}^\varepsilon) \overset{\text{Proposition 2}}{=} \mathbf{v}. \tag{95}$$

□

Proof of Proposition 5.

Because the error transformation $\mathbf{p} \mapsto \mathbf{p}^{\varepsilon} = \varphi^{\varepsilon}(\mathbf{p})$ is strictly monotonically increasing and defined on each component separately, it follows from **Lemma 1** that also the maps

$$p_C \to \pi^{\varepsilon}(\mathbf{p},\mathbf{q}) = \pi\Big(\varphi^{\varepsilon}\big((p_C, p_D)\big),\ \mathbf{q}\Big) \quad \text{and} \quad p_D \to \pi^{\varepsilon}(\mathbf{p},\mathbf{q}) = \pi\Big(\varphi^{\varepsilon}\big((p_C, p_D)\big), \mathbf{q}\Big) \tag{96}$$

are either strictly monotonically increasing, decreasing, or constant. The result then follows with the same argument as in the proof of **Proposition 3**, by replacing $\pi(\mathbf{p},\mathbf{q})$ with $\pi^{\varepsilon}(\mathbf{p},\mathbf{q})$. □

Proof of Theorem 3.

$(1) \Rightarrow (2)$. Because $\mathbf{q}$ is a generic Nash equilibrium, $\pi^{\varepsilon}(\mathbf{q},\mathbf{q}) \geq \pi^{\varepsilon}(\mathbf{p},\mathbf{q})$ for all generic strategies $\mathbf{p}$, which includes all reactive strategies.

$(2) \Rightarrow (3)$. By **Proposition 4**, we can compute the payoff $\pi^{\varepsilon}(\mathbf{q},\mathbf{q})$ of a memory-1 strategy $\mathbf{q}$ against itself by computing the payoff of its reactive marginalization $\tilde{\mathbf{q}}$ against itself, $\pi^{\varepsilon}(\tilde{\mathbf{q}},\mathbf{q})$. To this end, we use Eq. (27) to compute the entries of $\tilde{\mathbf{q}} = (\tilde{q}_C, \tilde{q}_D)$, yielding

$$\begin{aligned} \tilde{q}_C &= \frac{\varepsilon q_{CC} + (1-\varepsilon) q_{DC}}{1-(1-2\varepsilon)(q_{CC}-q_{DC})}, \\ \tilde{q}_D &= \frac{\varepsilon q_{CD} + (1-\varepsilon) q_{DD}}{1-(1-2\varepsilon)(q_{CD}-q_{DD})}. \end{aligned} \tag{97}$$

Similar to **Lemma 2**, we distinguish two cases, depending on whether or not this reactive marginalization is fully stochastic, that is whether or not $\tilde{\mathbf{q}} \in (0,1)^2$.

(*i*) $\tilde{\mathbf{q}}$ *is fully stochastic.* Because for reactive strategies $\mathbf{p} = (p_C, p_D)$ the maps $p_C \to \pi^{\varepsilon}(\mathbf{p},\mathbf{q})$ and $p_D \to \pi^{\varepsilon}(\mathbf{p},\mathbf{q})$ are either strictly monotonically increasing, decreasing, or constant (previous proof), it follows from $\tilde{\mathbf{q}} \in (0,1)^2$ and the assumption (33) that $\pi^{\varepsilon}(\mathbf{p},\mathbf{q})$ is constant for all reactive strategies $\mathbf{p}$. That is, $\varphi^{\varepsilon}(\mathbf{q})$ needs to satisfy Eq. (21). By applying the back-transformation (24), it follows that $\mathbf{q}$ needs to satisfy the conditions in Eq. (36).

(*ii*) $\tilde{\mathbf{q}}$ *is semi-stochastic or deterministic.* In this case, either $\tilde{q}_C \in \{0,1\}$ or $\tilde{q}_D \in \{0,1\}$. By Eq. (97) this implies

$$\begin{aligned} \tilde{q}_C \in \{0,1\} \quad &\Leftrightarrow \quad \mathbf{q} = (1, q_{CD}, 1, q_{DD}) \ \text{ or } \ \mathbf{q} = (0, q_{CD}, 0, q_{DD}) \\ \tilde{q}_D \in \{0,1\} \quad &\Leftrightarrow \quad \mathbf{q} = (q_{CC}, 1, q_{DC}, 1) \ \text{ or } \ \mathbf{q} = (q_{CC}, 0, q_{DC}, 0). \end{aligned} \tag{98}$$

We discuss each of these four cases in turn:

- $\mathbf{q} = (1, q_{CD}, 1, q_{DD})$. In this case, we can use the payoff formulas (31) and (32) to verify that $\pi^{\varepsilon}(\mathbf{q},\mathbf{q}) \geq \pi^{\varepsilon}(\mathit{TFT},\mathbf{q})$ if and only if

$$q_{DD} \leq \frac{(1-2\varepsilon)\,(b+\varepsilon c\, q_{CD}) - c}{(1-2\varepsilon)\,(b+\varepsilon c)}. \tag{99}$$

  On the other hand, an analogous computation shows that $\pi^{\varepsilon}(\mathbf{q},\mathbf{q}) \geq \pi^{\varepsilon}(\mathit{ALLC},\mathbf{q})$ if and only if the inequality in Eq. (99) is reversed. Together these two requirements imply the last condition in the characterization of partners (34). Finally, for strategies that satisfy $q_{CC} = q_{DC} = 1$ and the last condition in (34), both additional requirements $\pi^{\varepsilon}(\mathbf{q},\mathbf{q}) \geq \pi^{\varepsilon}(\mathit{ALLD},\mathbf{q})$ and $\pi^{\varepsilon}(\mathbf{q},\mathbf{q}) \geq \pi^{\varepsilon}(\mathit{ATFT},\mathbf{q})$ are met if and only if

$$q_{CD} \leq 1 - \frac{c}{(1-2\varepsilon)b}. \tag{100}$$

  Overall, we conclude that such a strategy $\mathbf{q} = (1, q_{CD}, 1, q_{DD})$ is robust with respect to deviations towards the four deterministic reactive strategies if and only if the additional conditions in (34) hold. In particular, such strategies are generic Nash equilibria, because robustness against all deterministic reactive strategies implies robustness against all generic strategies by **Propositions 4** and **5**.

- $\mathbf{q} = (0, q_{CD}, 0, q_{DD})$. Using the payoff formulas (31) and (32) one can show that $\pi^{\varepsilon}(\mathbf{q},\mathbf{q}) < \pi^{\varepsilon}(\mathit{ALLD},\mathbf{q})$ unless $q_{CD} = q_{DD} = 0$, that is $\mathbf{q} = \mathit{ALLD}$. We discuss the case of $\mathbf{q} = \mathit{ALLD}$ further below.

- $\mathbf{q} = (q_{CC}, 1, q_{DC}, 1)$. It is easy to show that $\pi^{\varepsilon}(\mathbf{q},\mathbf{q}) < \pi^{\varepsilon}(\mathit{ALLD},\mathbf{q})$ for all such $\mathbf{q}$.

- $\mathbf{q} = (q_{CC}, 0, q_{DC}, 0)$. If $\mathbf{q}$ is *ALLD*, then it is a Nash equilibrium (because defection is an equilibrium of the one-shot game). In the following let us thus assume that $q_{CC} > 0$ or $q_{DC} > 0$. In this case we obtain $\pi^{\varepsilon}(\mathbf{q},\mathbf{q}) \geq \pi^{\varepsilon}(\mathit{TFT},\mathbf{q})$ if and only if

$$q_{CC} \leq \frac{\varepsilon(1-2\varepsilon)c\, q_{DC} + c}{(1-2\varepsilon)(b+\varepsilon c)}. \tag{101}$$

  On the other hand, the requirement $\pi^{\varepsilon}(\mathbf{q},\mathbf{q}) \geq \pi^{\varepsilon}(\mathit{ALLD},\mathbf{q})$ is met if and only if the inequality in (101) is reversed. Together these two requirements imply the last condition in (35). Given this last condition and $q_{DD} = q_{CD} = 0$, it follows that $\pi^{\varepsilon}(\mathbf{q},\mathbf{q}) \geq \pi^{\varepsilon}(\mathit{ALLC},\mathbf{q})$ and $\pi^{\varepsilon}(\mathbf{q},\mathbf{q}) \geq \pi^{\varepsilon}(\mathit{ATFT},\mathbf{q})$ if and only if

$$q_{DC} \leq \frac{c}{(1-2\varepsilon)b}. \tag{102}$$

  We conclude that for strategies $\mathbf{q}$ that satisfy all conditions in (35) there are no profitable

deviations among the deterministic reactive strategies. Because of **Propositions 4** and **5**, this implies that there are no profitable deviations among the generic strategies, and hence $\mathbf{q}$ is a generic Nash equilibrium.

$(3) \Rightarrow (1)$ Follows immediately because partners and defectors are generic Nash equilibria by definition. Equalizers are generic Nash equilibria because any deviating player yields exactly the same payoff against an equalizer as the equalizer strategy yields against itself.

□

Supplementary References

[1] Hilbe, C., Martinez-Vaquero, L. A., Chatterjee, K. & Nowak, M. A. Memory-n strategies of direct reciprocity. *Proceedings of the National Academy of Sciences USA* **114**, 4715–4720 (2017).

[2] Nowak, M. A. & Sigmund, K. The alternating prisoner's dilemma. *Journal of Theoretical Biology* **168**, 219–226 (1994).

[3] Press, W. H. & Dyson, F. D. Iterated prisoner's dilemma contains strategies that dominate any evolutionary opponent. *PNAS* **109**, 10409–10413 (2012).

[4] Szolnoki, A. & Perc, M. Defection and extortion as unexpected catalysts of unconditional cooperation in structured populations. *Scientific Reports* **4**, 5496 (2014).

[5] Szolnoki, A. & Perc, M. Evolution of extortion in structured populations. *Physical Review E* **89**, 022804 (2014).

[6] Wu, Z.-X. & Rong, Z. Boosting cooperation by involving extortion in spatial prisoner's dilemma games. *Physical Review E* **90**, 062102 (2014).

[7] Hilbe, C., Hagel, K. & Milinski, M. Asymmetric power boosts extortion in an economic experiment. *PLoS ONE* **11**, e0163867 (2016).

[8] Sigmund, K. *The Calculus of Selfishness* (Princeton Univ. Press, Princeton, NJ, 2010).

[9] Akin, E. What you gotta know to play good in the iterated prisoner's dilemma. *Games* **6**, 175–190 (2015).

[10] Akin, E. The iterated prisoner's dilemma: Good strategies and their dynamics. In Assani, I. (ed.) *Ergodic Theory, Advances in Dynamics*, 77–107 (de Gruyter, Berlin, 2016).

[11] Hilbe, C., Traulsen, A. & Sigmund, K. Partners or rivals? Strategies for the iterated prisoner's dilemma. *Games and Economic Behavior* **92**, 41–52 (2015).

[12] Stewart, A. J. & Plotkin, J. B. Collapse of cooperation in evolving games. *Proceedings of the National Academy of Sciences USA* **111**, 17558 – 17563 (2014).

[13] Stewart, A. J. & Plotkin, J. B. From extortion to generosity, evolution in the iterated prisoner's dilemma. *Proceedings of the National Academy of Sciences USA* **110**, 15348–15353 (2013).

[14] Boerlijst, M. C., Nowak, M. A. & Sigmund, K. Equal pay for all prisoners. *American Mathematical Monthly* **104**, 303–307 (1997).

[15] Donahue, K., Hauser, O., Nowak, M. & Hilbe, C. Evolving cooperation in multichannel games. *Nature Communications* **11**, 3885 (2020).

[16] Selten, R. Reexamination of the perfectness concept for equilibrium points in extensive games. *International Journal of Game Theory* **4**, 25–55 (1975).

[17] Maynard Smith, J. *Evolution and the Theory of Games* (Cambridge University Press, Cambridge, 1982).

[18] Boyd, R. & Lorberbaum, J. No pure strategy is evolutionary stable in the iterated prisoner's dilemma game. *Nature* **327**, 58–59 (1987).

[19] van Veelen, M., García, J., Rand, D. G. & Nowak, M. A. Direct reciprocity in structured populations. *Proceedings of the National Academy of Sciences USA* **109**, 9929–9934 (2012).

[20] García, J. & van Veelen, M. In and out of equilibrium I: Evolution of strategies in repeated games with discounting. *Journal of Economic Theory* **161**, 161–189 (2016).

[21] García, J. & van Veelen, M. No strategy can win in the repeated prisoner's dilemma: Linking game theory and computer simulations. *Frontiers in Robotics and AI* **5**, 102 (2018).

[22] Boyd, R. Mistakes allow evolutionary stability in the repeated Prisoner's Dilemma game. *Journal of Theoretical Biology* **136**, 47–56 (1989).

[23] Lorberbaum, J. P., Bohning, D. E., Shastri, A. & Sine, L. E. Are there really no evolutionarily stable strategies in the iterated prisoner's dilemma? *Journal of Theoretical Biology* **214**, 155–169 (2002).

[24] Nowak, M. A. & Sigmund, K. Tit for tat in heterogeneous populations. *Nature* **355**, 250–253 (1992).

[25] Molander, P. The optimal level of generosity in a selfish, uncertain environment. *Journal of Conflict Resolution* **29**, 611–618 (1985).

[26] Nowak, M. A. & Sigmund, K. Invasion dynamics of the finitely repeated Prisoner's Dilemma. *Games and Economic Behavior* **11**, 364–390 (1995).

[27] Schmid, L., Chatterjee, K., Hilbe, C. & Nowak, M. A unified framework of direct and indirect reciprocity. *Nature Human Behaviour* **5**, 1292–1302 (2021).

[28] Nowak, M. A. & Sigmund, K. A strategy of win-stay, lose-shift that outperforms tit-for-tat in the Prisoner's Dilemma game. *Nature* **364**, 56–58 (1993).

[29] Dal Bó, P. & Fréchette, G. R. Strategy choice in the infinitely repeated prisoner's dilemma. *American Economic Review* **109**, 3929–3952 (2019).

[30] Pinheiro, F. L., Vasconcelos, V. V., Santos, F. C. & Pacheco, J. M. Evolution of all-or-none strategies in repeated public goods dilemmas. *PLoS Comput Biol* **10**, e1003945 (2014).

[31] Nowak, M. A. *Evolutionary dynamics* (Harvard University Press, Cambridge MA, 2006).

[32] Nowak, M. A. & May, R. M. Evolutionary games and spatial chaos. *Nature* **359**, 826–829 (1992).

[33] Killingback, T. & Doebeli, M. Spatial evolutionary game theory: Hawks and doves revisited.

Proceedings of the Royal Society B **263**, 1135–1144 (1996).

[34] Hauert, C. & Doebeli, M. Spatial structure often inhibits the evolution of cooperation in the snowdrift game. *Nature* **428**, 643–646 (2004).

[35] Perc, M., Gómez-Gardeñes, J., Szolnoki, A., Floría, L. M. & Moreno, Y. Evolutionary dynamics of group interactions on structured populations: A review. *Journal of The Royal Society Interface* **10**, 20120997 (2013).

[36] Brauchli, K., Killingback, T. & Doebeli, M. Evolution of cooperation in spatially structured populations. *Journal of Theoretical Biology* **200**, 405–417 (1999).

[37] Axelrod, R. & Hamilton, W. D. The evolution of cooperation. *Science* **211**, 1390–1396 (1981).

[38] Hauert, C. & Schuster, H. G. Effects of increasing the number of players and memory size in the iterated prisoner's dilemma: a numerical approach. *Proceedings of the Royal Society B* **264**, 513–519 (1997).

[39] Hilbe, C., Nowak, M. A. & Sigmund, K. The evolution of extortion in iterated prisoner's dilemma games. *Proceedings of the National Academy of Sciences USA* **110**, 6913–6918 (2013).

[40] Imhof, L. A. & Nowak, M. A. Stochastic evolutionary dynamics of direct reciprocity. *Proceedings of the Royal Society B* **277**, 463–468 (2010).

[41] Stewart, A. J. & Plotkin, J. B. The evolvability of cooperation under local and non-local mutations. *Games* **6**, 231–250 (2015).

[42] Wild, G. & Traulsen, A. The different limits of weak selection and the evolutionary dynamics of finite populations. *Journal of Theoretical Biology* **247**, 382–390 (2007).
