# Supplementary material for: Cooperation in alternating interactions with memory constraints
Source: Nat Commun. 2022 Feb 8;13:737. doi: 10.1038/s41467-022-28336-2 (PMC8825791; doi:10.1038/s41467-022-28336-2)
Supplement: Supplementary file 3 — Reporting Summary [file 41467_2022_28336_MOESM3_ESM.pdf]

nature research

Corresponding author(s): Christian Hilbe

Last updated by author(s): Jan 19, 2022

Reporting Summary

Nature Research wishes to improve the reproducibility of the work that we publish. This form provides structure for consistency and transparency in reporting. For further information on Nature Research policies, see Authors & Referees and the Editorial Policy Checklist .

Statistics

For all statistical analyses, confirm that the following items are present in the figure legend, table legend, main text, or Methods section.

n/a	Confirmed	
x	☐	The exact sample size (*n*) for each experimental group/condition, given as a discrete number and unit of measurement
x	☐	A statement on whether measurements were taken from distinct samples or whether the same sample was measured repeatedly
x	☐	The statistical test(s) used AND whether they are one- or two-sided<br>*Only common tests should be described solely by name; describe more complex techniques in the Methods section.*
x	☐	A description of all covariates tested
x	☐	A description of any assumptions or corrections, such as tests of normality and adjustment for multiple comparisons
x	☐	A full description of the statistical parameters including central tendency (e.g. means) or other basic estimates (e.g. regression coefficient) AND variation (e.g. standard deviation) or associated estimates of uncertainty (e.g. confidence intervals)
x	☐	For null hypothesis testing, the test statistic (e.g. *F*, *t*, *r*) with confidence intervals, effect sizes, degrees of freedom and *P* value noted<br>*Give P values as exact values whenever suitable.*
x	☐	For Bayesian analysis, information on the choice of priors and Markov chain Monte Carlo settings
x	☐	For hierarchical and complex designs, identification of the appropriate level for tests and full reporting of outcomes
x	☐	Estimates of effect sizes (e.g. Cohen's *d*, Pearson's *r*), indicating how they were calculated

Our web collection on statistics for biologists contains articles on many of the points above.

Software and code

Policy information about availability of computer code

Data collection	All individual-based simulations were run with Matlab 2019b. The baseline code is available online at http://dx.doi.org/10.17605/osf.io/v5hgd
Data analysis	All simulation results were analyzed and visualized with Matlab R2019b. Again, the respective code is available at http://dx.doi.org/10.17605/osf.io/v5hgd

For manuscripts utilizing custom algorithms or software that are central to the research but not yet described in published literature, software must be made available to editors/reviewers. We strongly encourage code deposition in a community repository (e.g. GitHub). See the Nature Research guidelines for submitting code & software for further information.

Data

Policy information about availability of data

All manuscripts must include a data availability statement. This statement should provide the following information, where applicable:

- Accession codes, unique identifiers, or web links for publicly available datasets
- A list of figures that have associated raw data
- A description of any restrictions on data availability

All data was generated with computer simulations. Source data for Figures 4, Figure 5, Figure 6a-f and Figure 6i are provided with this paper. Moreover, the raw data generated with the computer simulations, including the data that is necessary to create all figures is available at http://dx.doi.org/10.17605/osf.io/v5hgd

Field-specific reporting

Please select the one below that is the best fit for your research. If you are not sure, read the appropriate sections before making your selection.

Ecological, evolutionary & environmental sciences study design

All studies must disclose on these points even when the disclosure is negative.

Study description	This is a theoretical paper. All data is generated with computer simulations. The computer simulations are described in detail in the Methods section and in the Supplementary Information.<br>For this study, no empirical data was collected. In particular, there was no need to define a research sample, a sampling strategy, or a data collection scheme, nor was there a need to employ particular randomization or blinding procedures.
Research sample	This is a purely theoretical study and no empirical data was collected.
Sampling strategy	n/a (see Study description)
Data collection	n/a (see Study description)
Timing and spatial scale	n/a (see Study description)
Data exclusions	n/a (no data of any kind was excluded)
Reproducibility	n/a (see Study description)
Randomization	n/a (see Study description)
Blinding	n/a (see Study description)

Did the study involve field work? ☐ Yes ☒ No

Reporting for specific materials, systems and methods

We require information from authors about some types of materials, experimental systems and methods used in many studies. Here, indicate whether each material, system or method listed is relevant to your study. If you are not sure if a list item applies to your research, read the appropriate section before selecting a response.

Materials & experimental systems

n/a	Involved in the study	
☒	☐	Antibodies
☒	☐	Eukaryotic cell lines
☒	☐	Palaeontology
☒	☐	Animals and other organisms
☒	☐	Human research participants
☒	☐	Clinical data

Methods

n/a	Involved in the study	
☒	☐	ChIP-seq
☒	☐	Flow cytometry
☒	☐	MRI-based neuroimaging
